# Type I IFNs drive hematopoietic stem and progenitor cell collapse via impaired proliferation and increased RIPK1-dependent cell death during shock-like ehrlichial infection

**DOI:** 10.1371/journal.ppat.1007234

**Published:** 2018-08-06

**Authors:** Julianne N. P. Smith, Yubin Zhang, Jing Jing Li, Amanda McCabe, Hui Jin Jo, Jackson Maloney, Katherine C. MacNamara

**Affiliations:** Department of Immunology and Microbial Disease, Albany Medical College, Albany, New York, United States of America; University of California, Berkeley, UNITED STATES

## Abstract

Type I interferons (IFNα/β) regulate diverse aspects of host defense, but their impact on hematopoietic stem and progenitor cells (HSC/HSPCs) during infection remains unclear. Hematologic impairment can occur in severe infections, thus we sought to investigate the impact of type I IFNs on hematopoiesis in a tick-borne infection with a virulent ehrlichial pathogen that causes shock-like disease. During infection, IFNα/β induced severe bone marrow (BM) loss, blunted infection-induced emergency myelopoiesis, and reduced phenotypic HSPCs and HSCs. In the absence of type I IFN signaling, BM and splenic hematopoiesis were increased, and HSCs derived from *Ifnar1*-deficient mice were functionally superior in competitive BM transplants. Type I IFNs impaired hematopoiesis during infection by both limiting HSC/HSPC proliferation and increasing HSPC death. Using mixed BM chimeras we determined that type I IFNs restricted proliferation indirectly, whereas HSPC death occurred via direct IFNαR -mediated signaling. IFNαR-dependent signals resulted in reduced caspase 8 expression and activity, and reduced cleavage of RIPK1 and RIPK3, relative to *Ifnar1*-deficient mice. RIPK1 antagonism with Necrostatin-1s rescued HSPC and HSC numbers during infection. Early antibiotic treatment is required for mouse survival, however antibiotic-treated survivors had severely reduced HSPCs and HSCs. Combination therapy with antibiotics and Necrostatin-1s improved HSPC and HSC numbers in surviving mice, compared to antibiotic treatment alone. We reveal two mechanisms whereby type I IFNs drive hematopoietic collapse during severe infection: direct sensitization of HSPCs to undergo cell death and enhanced HSC quiescence. Our studies reveal a strategy to ameliorate the type I IFN-dependent loss of HSCs and HSPCs during infection, which may be relevant to other infections wherein type I IFNs cause hematopoietic dysfunction.

## Author summary

The *Ehrlichiae* are important emerging, tick-borne pathogens that cause immune suppression and cytopenias, though the underlying mechanisms are unclear. In a model of shock-like illness caused by *Ixodes ovatus* ehrlichia, type I interferons (IFNs) induce hematopoietic dysfunction by reducing hematopoietic stem cell (HSC) proliferation and driving cell death of hematopoietic progenitors (HSPCs). Using mixed bone marrow chimeras, we demonstrate that HSPC loss occurs via intrinsic type I IFN signaling, whereas HSC proliferation is regulated via an extrinsic mechanism. In contrast to sterile inflammation, infection-induced type I IFNs induced RIPK1-dependent loss of hematopoietic progenitors. HSPCs were rescued during infection by inhibiting RIPK1 with Necrostatin-1s. While antibiotic treatment protected against otherwise lethal infection, mice recovering from infection exhibited significantly reduced HSCs and HSPCs. Co-treatment with both antibiotics and Necrostatin-1s significantly increased HSPC frequencies and the number of HSCs compared to antibiotics alone. Blood production is essential for life and necessary for host defense, thus our work reveals a therapeutic strategy to rescue and improve hematopoiesis in patients recovering from serious infectious disease.

## Introduction

Acute infection induces demand-adapted hematopoiesis, characterized by increased hematopoietic stem cell and progenitor cell (HSC and HSPC) proliferation, to support production and mobilization of immune cells or platelets [1–5]. Infection induced ‘emergency myelopoieisis’ results in increased production of effector myeloid cells that promote bacterial clearance [3, 6]. However, excessive proliferation of HSCs and HSPCs can lead to functional impairment and induce hematopoietic suppression [7–10],[11], though the precise mechanisms driving HSC/HSPC impairment have only recently been investigated [3, 12–15]. The *Ehrlichiae* are emerging tick-borne pathogens that cause an acute, febrile disease called human monocytic ehrlichiosis (HME) [16]. *Ehrlichia* are obligate, intracellular alpha-proteobacteria of the *Anaplasmataceae* family, and contain gram-negative cell wall structures but lack the genes that encode lipopolysaccharide and peptidoglycan [17, 18]. HME disease severity can vary greatly, and in some cases life-threatening complications include multi-organ failure similar to septic shock syndrome [19]. *Ixodes ovatus* ehrlichia (IOE) is a highly virulent strain that causes shock-like illness in mice [20, 21], and is therefore an ideal model to study fatal HME [22]. Vector borne diseases are increasing, and current vaccines are lacking [23], therefore, acute and chronic sequelae induced by tick-borne infections are clinically significant and represent a growing health care concern. HSCs are essential for lifelong hematopoiesis and supply all cells necessary for hemostasis, immunity, and oxygenation, thus delineating the mechanisms that impact HSC function during acute infection is important for our full understanding of infection-induced pathology.

Type I interferons (IFNα/β) are induced in response to nearly all infections. IFNα receptor (IFNαR) signaling stimulates diverse immune cell effector functions, and IFNα/β regulate HSCs directly and through the bone marrow (BM) microenvironment [24, 25]. However, it is currently unclear how type I IFNs regulate HSC function during infection. Sterile IFNα/β stimulation induces HSC proliferation, caspase activation, and apoptosis [8]. HSPCs from IFNα-treated patients do not exhibit apoptotic priming [26], however, and IFNα/β promote hematopoietic precursor survival in a murine model of opportunistic lung infection [27]. Therefore, type I IFNs can have diverse impacts on HSC function. HSCs sustain immune function over the lifetime of an organism, and must be maintained through multiple rounds of inflammatory stress, thus clarifying the impact of IFNα/β on hematopoiesis during infection and disease has important implications.

IFNα/β promote lethal, shock-like pathology in mice infected with the ehrlichial pathogen *Ixodes ovatus* ehrlichia (IOE) [28, 29]. IOE-induced murine ehrlichiosis is pathologically similar to Rocky Mountain spotted fever, characterized by severe liver damage, leukocyte necrosis, and bone marrow (BM) hypoplasia [30]. Early antibiotic treatment is essential for host survival [31], and loss of *Il18ra*, *Tnfr1/2*, or *Nod2* mitigates IOE-induced immunopathology and prolongs survival but does not prevent mortality [21, 32, 33]. Full protection from IOE-induced mortality has only been identified in mice lacking *Ifnar1* or *Tlr9* [28, 29, 34], and mechanisms of pathogenesis are not well defined.

Type I IFNs mediate host defense against viral infection, but are also involved in immunopathology, including BM hypocellularity and impaired immunity in a number of bacterial infections [11]. Type I IFNs drive receptor interacting serine/threonine protein kinase 3 (RIPK3)-dependent macrophage necroptosis in *Salmonella typhimurium* infection [35], and are required for TNFα-induced systemic inflammatory response syndrome [36], wherein RIPK3 drives pathology [37]. In a shock-like infection induced by a virulent ehrlichial pathogen, IFNα/β-induced BM failure and hematopoietic suppression via increased RIPK1 activation and diminished caspase 8 expression. HSPC depletion and cell death depended upon cell-intrinsic IFNαR expression, whereas enforced HSC quiescence also depended on IFNαR, but was not due to direct signaling in HSCs. Together, impaired proliferation and increased cell death contributed to hematopoietic collapse during severe infection and reduced HSC and HSPC recovery following infection.

## Results

### Type I IFNs impair hematopoiesis during acute ehrlichial infection

**Fig 1:**
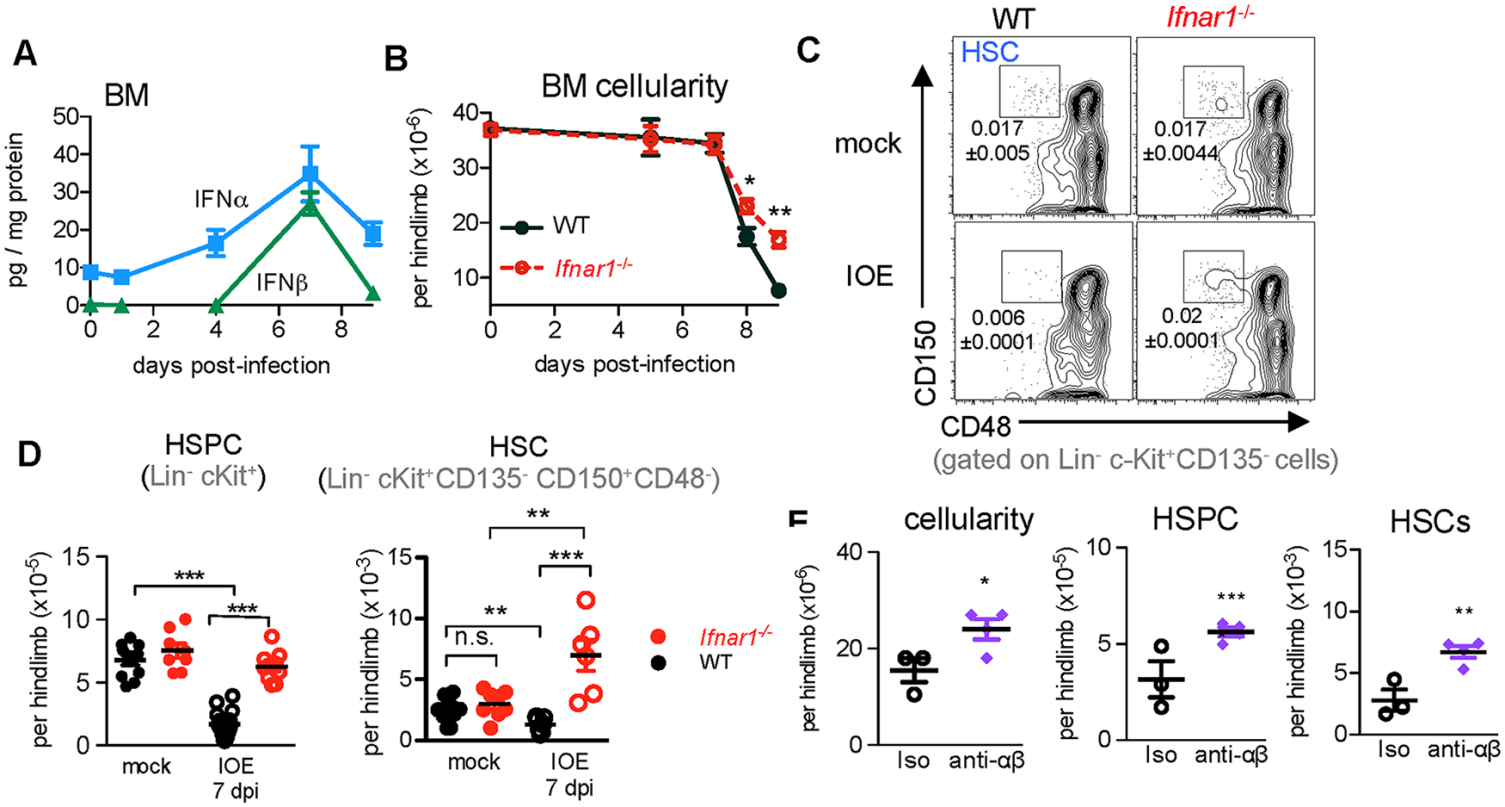
HSC and HSPC depletion in IOE infection is type I IFN-dependent. **(A)** BM IFNα and IFNβ levels at indicated days post-infection (d.p.i.) of wildtype (WT) mice. n = 4–10 mice/group. **(B)** BM cellularity of infected WT and *Ifnar1*^-/-^ mice. n = 4–14 mice/group, *P* < 0.05 for *Ifnar1*^-/-^ vs. WT by 2-way ANOVA. Significant differences were observed between strains on days 8 and 9 post-infection. **(C)** Representative flow cytometric plots of CD150 and CD48 expression among Lin^-^ cKit^+^ CD135^-^ BM cells from mock and IOE infected WT and *Ifnar1*^-/-^ mice. Gated population represents phenotypic HSCs, and numbers indicate absolute frequencies of HSCs. (**D**) Numbers of HSPCs (Lin^-^ cKit) and HSCs (Lin^-^ cKit^+^ CD135^-^ CD150^+^ CD48^-^) in WT and *Ifnar1*^-/-^ mice, n = 5–10 mice/group, **P < 0.01, ***P < 0.001 **(E)** BM cellularity, HSPCs, and HSCs in isotype- or anti-αβ–treated mice in IOE infected mice at 8 d.p.i. n = 3–4 mice/group.

IOE infection elicits robust production of IFNα/β, which is detected in the serum, spleen, and liver of wild type (WT) mice [28, 29], as well as in the BM (Fig 1A). IOE infection induces BM hypocelluarity with a profound decrease in cellularity on day 8 post-infection, which is mitigated by *Ifnar1* deletion (Fig 1B). IFNαR-dependent signals also impaired myeloid progenitor cell activity in the BM during infection (S1A Fig), and significantly enhanced monocyte differentiation was observed in cultured Lin^-^ cells from *Ifnar1*^-/-^ mice, relative to wild type mice (S1B Fig). HSPCs can mobilize from the BM and engraft the spleen during systemic inflammation [38], though neither WT nor *Ifnar1*^-/-^ mice showed evidence of HSPC mobilization in the peripheral blood (S1C Fig), and WT mice showed little to no induction of splenic extramedullary hematopoiesis (EMH). In contrast, robust EMH was observed in the spleens of *Ifnar1*^*-/-*^ mice (S1D–S1G Fig), corresponding to increased mature myeloid cells (S1H–S1K Fig), demonstrating IFNα/β suppress hematopoiesis systemically during IOE infection.

HSPCs are heterogeneous, proliferative and consist of lineage-biased progenitors important for daily blood production. The HSPC pool requires replenishment by HSCs, which typically reside in a dormant state in the BM. We examined HSPCs and HSCs on day 7 post-infection, when we detect type I IFNs in the BM and the day prior to the striking decrease in cellularity. At this time, both HSPCs (Lineage^-^, c-Kit^+^) and phenotypic long-term HSCs (Lineage^-^ c-Kit^+^ CD135^-^ CD150^+^ CD48) were depleted from the BM of WT mice during infection, but in *Ifnar1*^-/-^ mice HSPCs were maintained and HSCs underwent a significant expansion as determined by both frequency and number (Fig 1C and 1D). Neutralization of IFNα/β during infection of WT mice resulted in improved BM cellularity and significant protection against infection-induced loss of HSPCs and HSCs relative to isotype control treated mice (Fig 1E), similar to observations in *Ifnar1*^-/-^ mice.

**Fig 2:**
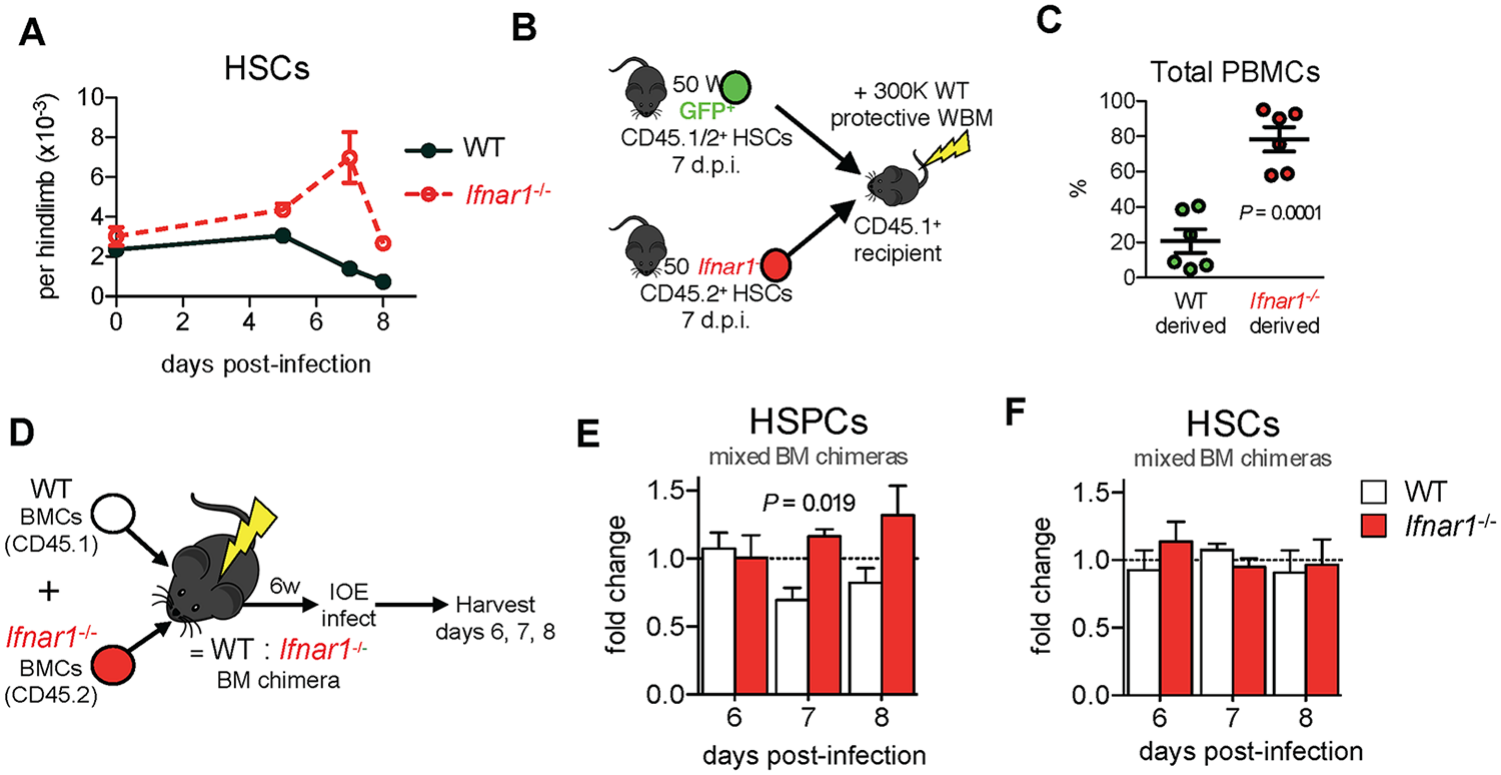
IFNα/β reduce phenotypic and functional HSCs and HSPCs via direct and indirect mechanisms. **(A)** HSCs, analyzed using the strategy in Fig 1C over time in WT and *Ifnar1*^-/-^ mice, n = 4–14 mice/group, *P* < 0.0001 for *Ifnar1*^-/-^ vs. WT by 2-way ANOVA. **(B)** Transplantation strategy used to test HSC function in IOE-infected WT and *Ifnar1*^-/-^ mice. Equal numbers of HSCs (50) were sort purified and competitively transferred to irradiated recipients. **(C)** Repopulation of total peripheral blood mononuclear cells (PBMCs) by WT and *Ifnar1*^-/-^ HSCs 16 weeks post-transplant. n = 6 recipient mice. **(D)** Generation of radiation-induced WT:*Ifnar1*^-/-^ mixed BM chimeric mice. (**E-F**) Fold change in proportions of WT to *Ifnar1*^*-/-*^ HSPCs and HSCs in infected WT:*Ifnar1*^*-/-*^ chimeric mice 6–8 d.p.i., normalized to baseline chimerism. n = 3 mice/time point.

Infection of *Ifnar1*^-/-^ mice resulted in a striking increase in phenotypic HSCs (Fig 2A). To address whether their phenotype corresponded to function we performed transplantation assays. In the absence of infection, IFNα/β signaling did not impact HSPC repopulating activity in transplantation (S2A Fig). However, sort-purified HSCs derived from IOE-infected *Ifnar1*^-/-^ mice were functionally superior to those derived from WT mice as evidenced by increased contribution to blood production and reconstitution of both lymphoid and myeloid compartments (Fig 2B and 2C and S2B Fig). HSCs derived from IOE-infected WT mice exhibited a loss of lymphoid potential, demonstrating reduced stem cell function. Thus, the decline in HSC number and function during shock-like infection was dependent on IFNα/β, and may, in part, be due to the intense demand for and loss of HSPCs.

We next generated WT:*Ifnar1*^-/-^ mixed BM chimeric mice (Fig 2D) to evaluate the direct, or intrinsic, impact of IFNαR-mediated signals on HSPCs and HSCs during infection. Within the HSPC compartment of IOE-infected chimeric mice we observed an increase in *Ifnar1*^-/-^ cells relative to WT counterparts (Fig 2E), indicating that HSPCs are directly impaired by IFNα/β during infection. However, within the HSC compartment we observed a similar fold change in WT and *Ifnar1*^-/-^ cells demonstrating IFNα/β did not directly affect HSC numbers (Fig 2F). This finding suggests that a soluble factor, dependent on IFNαR-signaling, likely mediates the loss of HSCs in WT mice.

### IFNα/β impair HSPC proliferation and maintain HSC quiescence during IOE infection

**Fig 3:**
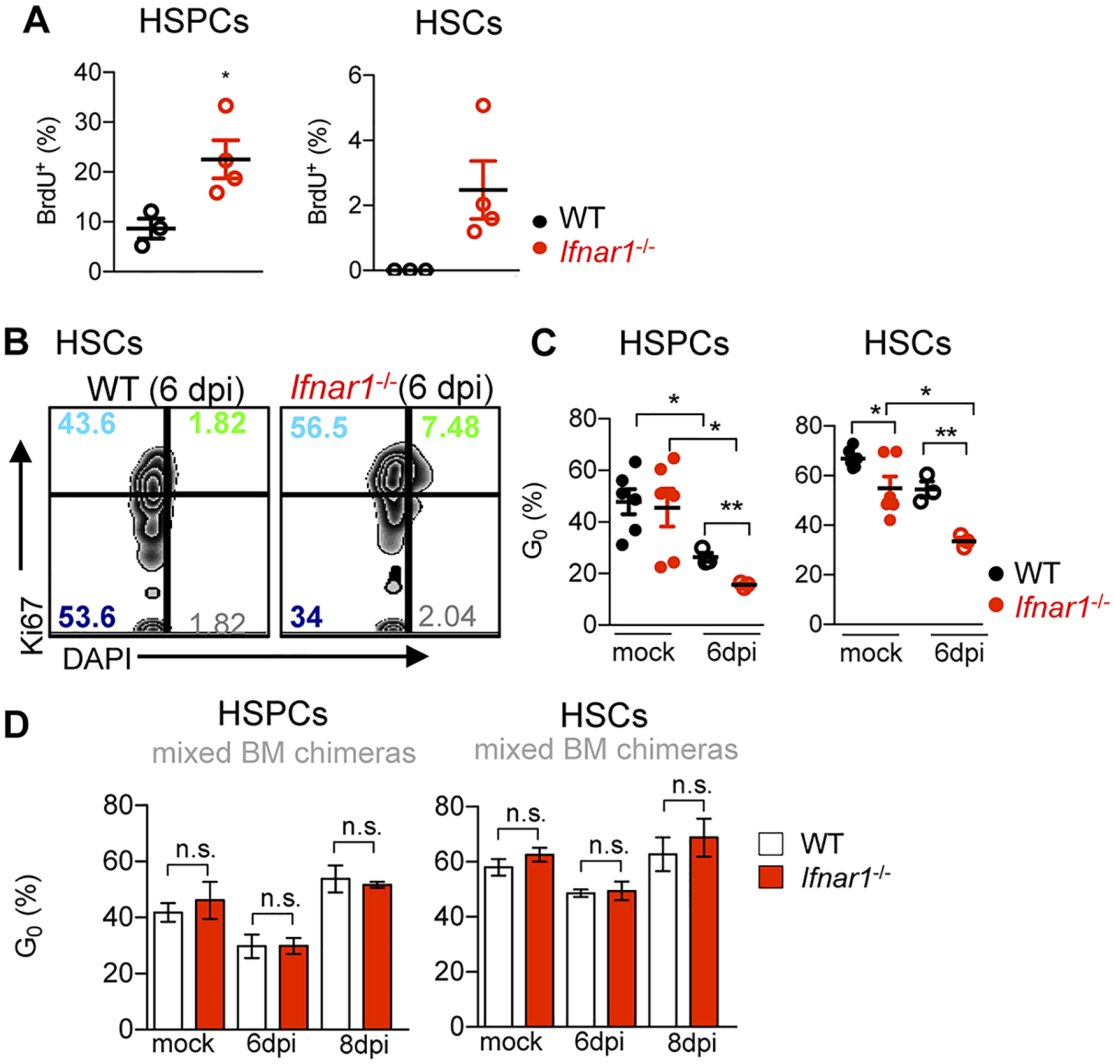
IFNα/β impair infection-induced proliferation of HSCs and HSPCs and indirectly restrict cycling. **(A)** 4-hour BrdU incorporation by HSPCs and HSCs in IOE-infected WT and *Ifnar1*^-/-^ mice 7 d.p.i. **(B)** Representative plots of Ki67 and DAPI staining, gated on HSCs from infected WT and *Ifnar1*^-/-^ mice 6 d.p.i. Numbers indicate quadrant percentages. (**C**) Percent cells in G_0_ among HSPCs and HSCs in mock and IOE-infected WT and *Ifnar1*^-/-^ mice 6 d.p.i. (**D**) The percent of WT and *Ifnar1*^-/-^ cells in G_0_ in mock- or IOE-infected WT: *Ifnar1*^*-/-*^ chimeric mice (as described in Fig 2D) at 6 and 8 d.p.i. *P < 0.05, **P < 0.001.

IFNα/β induce HSC proliferation during sterile inflammation [8, 39, 40]. We reasoned that loss of HSCs and HSPCs during IOE infection may be driven by enhanced and unsustainable IFNαR-dependent proliferation. Our data did not support this, however, as HSPCs and HSCs were more proliferative in *Ifnar1*^-/-^ mice, exhibiting enhanced incorporation of BrdU during IOE infection, relative to WT mice (Fig 3A). HSCs are typically in G_0_, though HSC quiescence was reduced in *Ifnar1*^-/-^ mice at both steady state and upon infection (Fig 3B and 3C) indicating that HSCs proliferated more in *Ifnar1*^-/-^ mice whereas WT HSCs remained quiescent. Infection caused a reduction in the proportion of G_0_ HSPCs in both WT and *Ifnar1*^-/-^ mice, though a more marked reduction was observed in the absence of type I IFNs. Because the inflammatory milieu varies between WT and *Ifnar1*^*-/-*^ mice (S3 Fig), we evaluated cell cycle regulation within the same microenvironment using WT:*Ifnar1*^-/-^ mixed BM chimeric mice. When in the same microenvironment, WT and *Ifnar1*^-/-^ HSPCs and HSCs showed similar cycling at both steady state and during infection (Fig 3D). Thus, the infection-induced difference in proliferation of HSCs and HSPCs did not depend on direct IFNαR-signaling, and was due to an extrinsic or soluble factor. Our data suggest that increased HSCs and HSPCs in *Ifnar1*^-/-^ mice may result from their enhanced proliferation and reduced quiescence, relative to WT mice.

### IFNα/β mediated HSPC cell death

HSPCs undergo type I IFN-dependent p53-mediated apoptosis in models of sterile, polyI:C (pI:C)-driven inflammation [8, 40], suggesting type I IFNs may drive infection-induced hematopoietic failure via increased cell death of HSPCs. To model IOE infection-induced IFNα/β exposure, we repeatedly administered endotoxin-free pI:C to WT and *Ifnar1*^-/-^ mice, which caused transient BM hypocellularity, and an IFNαR-dependent reduction in HSCs and HSPCs (S4A–S4D Fig). Type I IFNs were also required for the pI:C-induced increase in dead and dying Lin^-^ CD48^-^ cells (enriched for long- and short-term HSCs; S4E Fig;[41]. Consistent with prior reports that pI:C induces apoptosis, we observed increased numbers of cells that bound fluorescent probes for active caspases (caspase 3/7 and 8; S4F Fig). Thus, during sterile inflammation IFNα/β-dependent depletion of HSPCs correlates with induction of caspase activity and sensitization to apoptosis.

**Fig 4:**
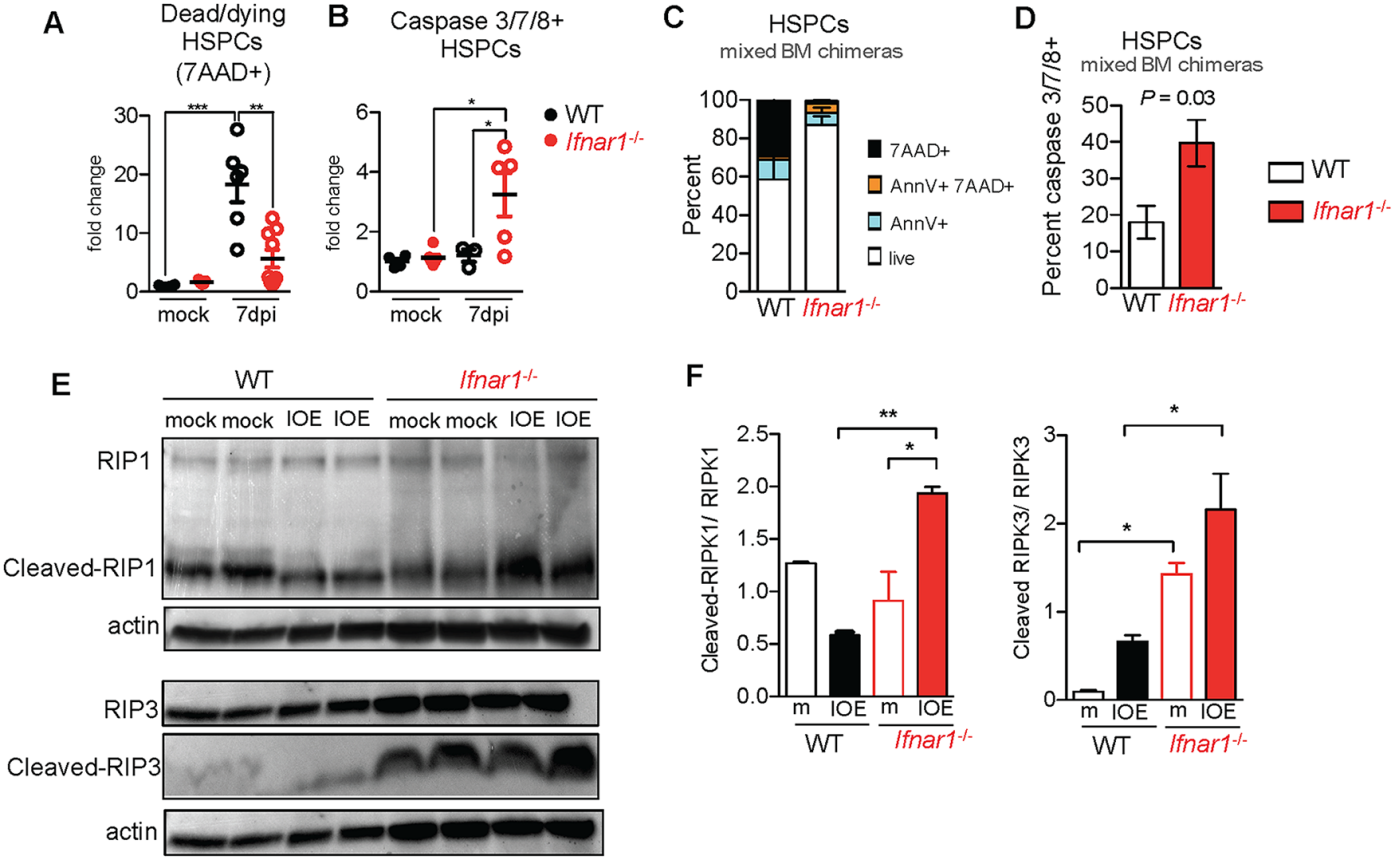
Type I IFNs induce caspase-independent HSPC death during IOE infection. **(A)** Fold change in dead/dying cell frequency within the CD48^-^ HSPC population of WT and *Ifnar1*^-/-^ mice 7 d.p.i. normalized to WT mock levels. n = 5–9 mice/group. **(B)** Fold change in the proportion of live HSPCs with active caspases 8 or 3/7 over mock mice. n = 3–6 mice/group. **(C)** Frequency of apoptotic (Annexin V+ 7AAD-), dying (Annexin V+ 7AAD+), and dead (Annexin V- 7AAD+) cells and (**D**) caspase 8 or 3/7-active cells (gated on live, 7AAD- cells) among WT and *Ifnar1*^-/-^ HSPCs in mixed BM chimeric mice 7 d.p.i. n = 3 mice. (**E**) Detection of full length RIP1, cleaved RIP1, full length RIP3, cleaved RIP3, and actin in BM lysates of WT and *Ifnar1*^-/-^ mock and IOE–infected mice 7 d.p.i. Lanes represent individual mice. (**F**) Fold change of cleaved RIP1 to full length RIP1 and cleaved RIP3 to full length RIP3 in WT and *Ifnar1*^-/-^ mock (m) and IOE–infected mice, 7 d.p.i. *P < 0.05, **P < 0.001, ***P < 0.0001.

Similar to sterile inflammation achieved by pI:C injection, IOE infection induced an IFNαR-dependent loss in HSCs and HSPCs. To assess whether IOE infection induced apoptosis we evaluated Annexin V staining, and found increased Annexin V+ HSPCs in response to IOE infection in WT, but not *Ifnar1*^-/-^ mice (S5A and S5B Fig). More striking was the nearly twenty-fold increase in the proportion of dead and dying cells (7-AAD+) among the HSPC pool in WT mice, whereas *Ifnar1*^-/-^ mice exhibited no such increase at day 7 post-infection (Fig 4A). In contrast to pI:C exposure, enhanced caspase 3/7/8-positive cells were not observed in IOE-infected WT mice (Fig 4B). Rather, we noted a significant increase in caspase 3/7/8-positive cells in IOE infected *Ifnar1*^-/-^ mice. To determine if direct IFNα/β signaling sensitized HSPCs to die, we evaluated viability and caspase activity in WT:*Ifnar1*^*-/-*^ mixed BM chimeric mice. A striking and direct IFNα/β-dependent increase in dead (7AAD+) and apoptotic (Annexin V+) progenitors was observed 7 d.p.i. (Fig 4C). WT cells were not caspase 3/7/8-positive, whereas a significant increase in caspase 3/7/8-positive HSPCs was observed among *Ifnar1*^-/-^ cells in the same mice (Fig 4D). These data establish that IFNα/β deplete HSPCs directly during infectious inflammation, and suggest IFNα/β may sensitize progenitors to undergo caspase-independent necroptotic cell death during IOE infection.

Caspase 8 and FADD are effectors of apoptosis that can also block necroptosis by cleaving RIP1 and RIP3 [42], suggesting improved HSC/HSPCs in *Ifnar1*^-/-^ mice may be due to distinct modes of cell death. In support of this, we observed enhanced RIPK1 cleavage in BM of IOE-infected *Ifnar1*^-/-^ mice, relative to infected WT mice (Fig 4E and 4F). At the same time, we observe a clear RIPK3 cleavage product in the BM of *Ifnar1*^-/-^ mice, and significantly reduced cleavage of RIPK3 in WT mice. Thus, in *Ifnar1*^-/-^ mice increased caspase-3/7/8-positive cells correlated with enhanced cleavage of key effectors of necroptotic and apoptotic signaling.

TNFα induces pathology in IOE infection [21], and TNFα is a driver of necroptotic cell death, a form of programmed necrosis involving RIPK1, RIPK3, and the effector protein MLKL [43]. While IFNα/β can stimulate TNFR2 expression in some cellular contexts [44], we observed similar BM TNFα levels and equivalent TNFR2 expression by progenitor cells in IOE-infected WT and *Ifnar1*^-/-^ mice (S6A and S6B Fig). These results suggest differences in TNF signaling do not account for the differences observed in WT and *Ifnar1*^-/-^ mice. To investigate a role for type I IFNs in necroptosis we next examined RIPK1 and RIPK3 in sort-purified HSPCs derived from IOE-infected mice. In whole BM we found similar expression of RIPK3 and MLKL in WT and *Ifnar1*^*-/-*^ mice during IOE infection (S6C and S6D Fig). Immunostaining and confocal imaging of RIPK1 and RIPK3 in sort-purified HSPCs revealed distinct IFNαR-dependent changes during IOE infection. RIPK1 intensity was greater in WT mice, relative to *Ifnar1*^*-/-*^ mice (S7A Fig), and we noted increased colocalized RIPK3 and RIPK1 signals in the cytoplasm of HSPCs derived from infected WT mice, relative to *Ifnar1*^*-/-*^ mice (S7B Fig), suggestive of RIPK1 and RIPK3 interaction.

### IFNα/β limit caspase 8-induced survival of HSPCs in IOE-infected mice

**Fig 5:**
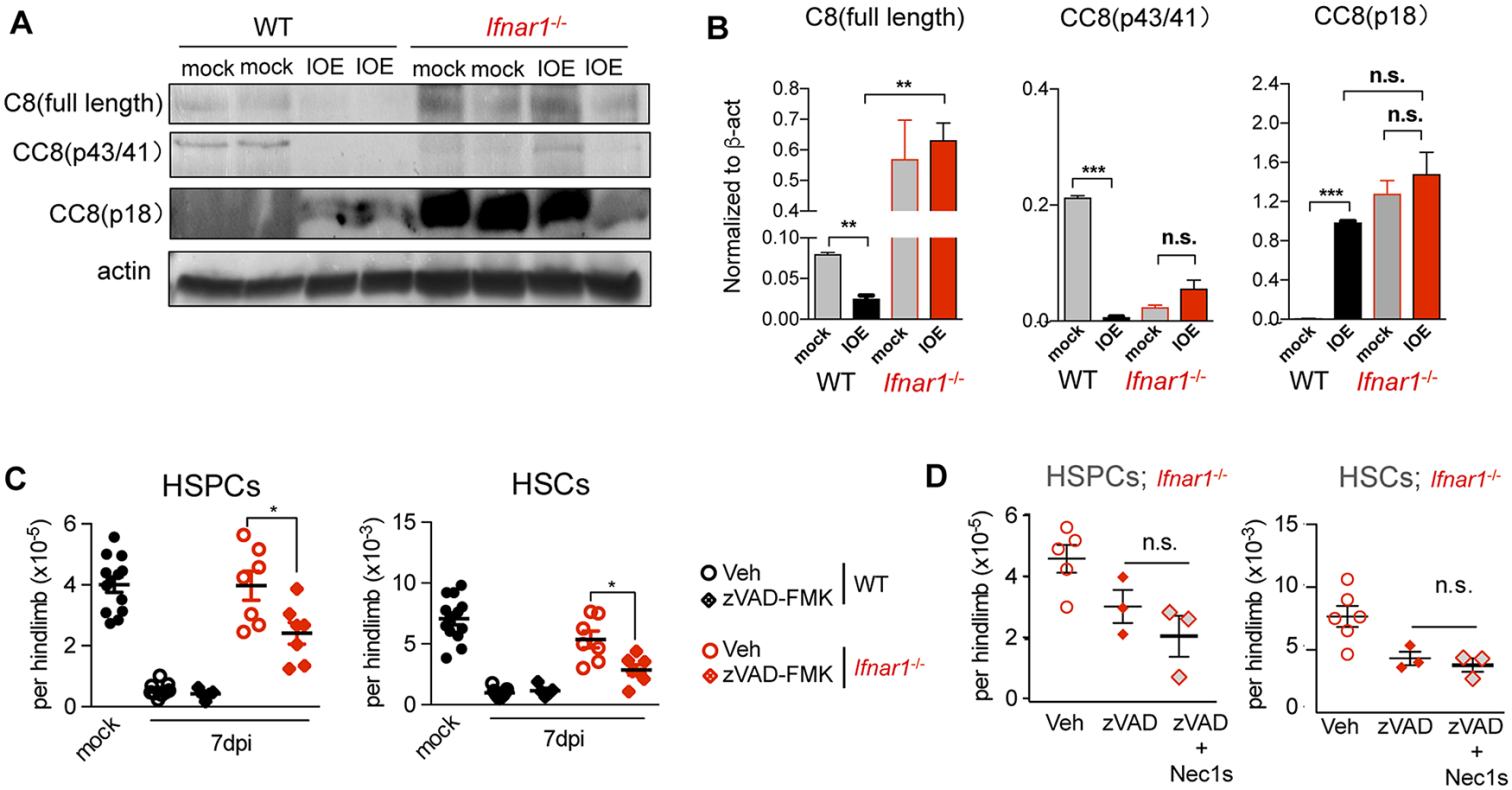
IFNαR-dependent regulation of caspase 8 during IOE infection. **(A)** Detection of full length (C8), and cleaved caspase 8 (CC8) and β-actin in BM cell lysates from mock and infected WT and *Ifnar1*^-/-^ mice 7d.p.i. Lanes represent individual mice. **(B)** Relative amounts of C8 and CC8 p41/43 and CC8 p18 in WT and *Ifnar1*^-/-^ mock and IOE–infected mice 7 d.p.i., normalized to β-actin. **(C)** HSPCs and HSCs in mock and vehicle (veh) control- or zVAD-FMK-treated infected WT and *Ifnar1*^-/-^ mice 7 d.p.i. n = 5–13 mice/group. **(D)** Vehicle-, zVAD-FMK-treated, or zVAD-FMK- and Necrostatin-1s treated *Ifnar1*^-/-^ mice day 7 p.i. n = 3–5 mice/group. *P < 0.05, **P < 0.001, ***P < 0.0001.

The increase in caspase-active progenitors in *Ifnar1*^*-/-*^ mice detected by flow cytometry coincided with enhanced caspase 8 expression in the BM, including full length and the p41/43 and p18 cleavage products, all of which were less abundant in WT IOE-infected mice (Fig 5A and 5B). The striking increase in caspase 8 in *Ifnar1*^*-/-*^ mice as well as increased FADD expression (S6E Fig), suggest protection of HSPCs in *Ifnar1-/-* mice may rely on caspase activation. To evaluate this we treated infected WT and *Ifnar1*^-/-^ mice with the pan-caspase inhibitor zVAD-FMK (zVAD). In WT mice, zVAD did not alter HSPC or HSC depletion, consistent with our observation that WT HSPCs do not exhibit caspase activity, and confirm that HSPC depletion in WT mice is independent of caspase activity. However, zVAD significantly reduced HSPCs and HSCs in IOE-infected *Ifnar1*^-/-^ mice (Fig 5C). To test whether zVAD licensed necroptotic cell death of HSPCs, we administered a specific inhibitor of RIPK1 kinase activity, Necrostatin-1s (Nec-1s), [45]. In contrast to our prediction that caspase inhibition induced RIPK1-dependent cell death, Nec-1s did not rescue the z-VAD-induced loss of HSPCs in *Ifnar1-/-* mice (Fig 5D). These findings suggest a complex role for caspase activity in protection of HSPCs and HSCs, and provide evidence that in the context of global *Ifnar1*-deficiency caspase activity is protective to HSC/HSPCs independently of RIPK1-dependent cell death. In addition to regulating cell death, caspase 8 can induce survival and proliferation signals in lymphocytes and primitive hematopoietic progenitors [46–48], suggesting the profoundly increased caspase 8 expression observed in *Ifnar1-/-* mice may induce pro-survival and/or proliferative signals that protect and increase the pool of HSCs and HSPCs during IOE infection.

### An intrinsic role for RIPK3 activity in infection-induced HSPC depletion

In the absence of caspase 8 activity, RIPK1-RIPK3 interactions promote RIPK3-dependent phosphorylation of the terminal necroptosis effector, MLKL [49], and can also drive cell death via NLRP3 inflammasome activation [50]. Unlike *Ifnar1*^*-/-*^ mice, *Caspase1*^*-/-*^ and *Nlrp3*^*-/-*^ mice were not protected from IOE infection [28], thus we sought to further address a potential role for necroptotic cell death in driving HSPC loss during IOE infection.

**Fig 6:**
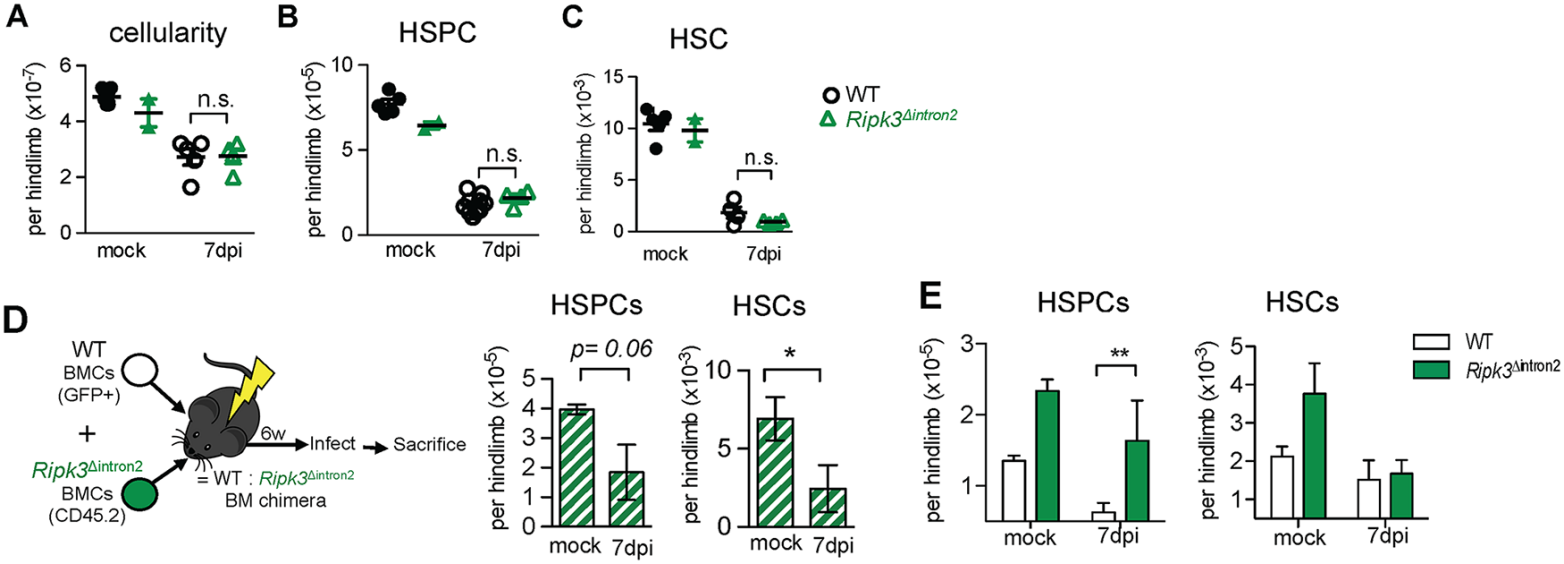
Targeting RIPK3 kinase activity protects HSPCs from IOE infection-induced depletion in mixed BM chimeric mice. **(A-C)** BM cellularity (A), HSPCs (B), and HSCs (C) in mock or infected WT and *Ripk3*^Δintron2^ mice 7 d.p.i. n = 4–5 mice/infected group; n = 2–4 mice/mock group. (**D**) Generation and treatment of *Ripk3*^Δintron2^:WT mixed BM chimeric mice, and numbers of HSPCs and HSCs in mock and IOE-infected chimeric mice 7 d.p.i. (**E**) Numbers of WT (white bars) and *Ripk3*^Δintron2^ (green bars) HSPCs and HSCs in mock and IOE-infected chimeric mice 7 d.p.i. n = 5–6 mice/group.*P < 0.05, **P < 0.01.

Mice with a mutation in the kinase domain of RIPK3 (*Ripk3*^Δintron2^), which is necessary for phosphorylation of MLKL, exhibited similar cellularity and HSPC and HSC numbers as IOE-infected WT mice (Fig 6A–6C). RIPK3 regulates type I IFN production during influenza infection ([51], however, we observed normal production of type I IFNs in response to IOE infection in *Ripk3*^Δintron2^ mice (S8A Fig). RIPK3 has two functional domains: an N-terminal serine-threonine kinase, and a C-terminal RIP homotypic interaction motif (RHIM). While kinase activity is required for necroptosis, the RHIM domain has additional functions, including promoting NF-kB signaling and cytokine production [52]. We confirmed that the RHIM domain was intact in *Ripk3*^Δintron2^ mice using an antibody against the C-terminus of RIPK3, though RIPK3 expression is reduced in *Ripk3*^Δintron2^ mice (S8B Fig). Because RIPK3 can drive apoptosis under some conditions [53, 54] we examined cell death in infected *Ripk3*^Δintron2^ mice. Compared to WT mice, *Ripk3*^Δintron2^ mice exhibited increased apoptosis (Annexin V+ cells; S8C Fig), suggesting HSPC loss in the absence of RIPK3 favors apoptosis. Nec-1s treatment did not improve HSPC or HSC frequencies in *Ripk3*^Δintron2^ mice (S8D Fig).

As type I IFNs reduced HSCs via an extrinsic factor, we reasoned that RHIM-dependent functions of RIPK3 potentially obscured a protective in *Ripk3*^Δintron2^ mice. To address this possibility, we generated mixed BM chimeric mice with *Ripk3*^Δintron2^ BM (WT: *Ripk3*^Δintron2^; Fig 6D). Within the HSPC and HSC populations, we observed a greater proportion of *Ripk3*^Δintron2^ cells relative to WT cells, even in the absence of infection, suggesting RIPK3 impairs HSC and HSPCs in the context of transplantation (Fig 6E). In response to infection, the proportion of *Ripk3*^Δintron2^ HSPCs was increased relative to WT cells, indicating a cell-intrinsic role for RIPK3 in the infection-induced loss of HSPCs. However, equal proportions of WT and RIPK3 HSCs were observed after infection, demonstrating intrinsic RIPK3 kinase activity does not mediate HSC loss. These data are consistent with our observations in WT: *Ifnar1*^*-/-*^ chimeras and suggest RIPK3 activation is downstream of type I IFN signals, as observed in macrophages [55]. Our data suggest that type I IFNs induce intrinsic, RIPK3-mediated death of HSPCs.

### RIPK1 activity promotes HSPC death and drives HSC loss during infection

**Fig 7:**
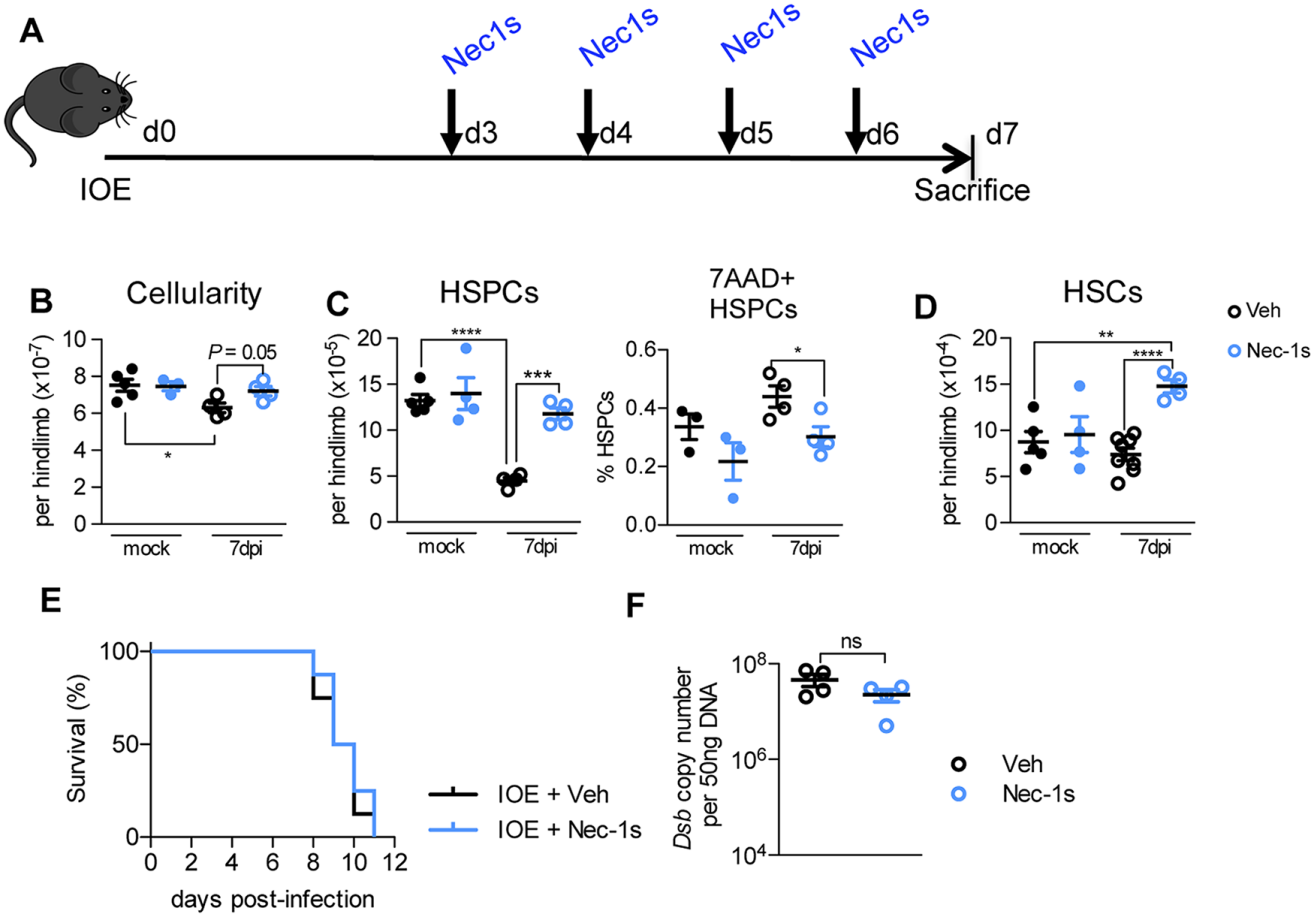
RIPK1 antagonism during infection rescues HSPCs and HSCs but does not impact bacterial burden or survival. **(A)** Schematic of IOE infection and treatment with Necrostatin 1 (Nec1s), which was administered twice/day. **(B-D)** BM cellularity (B), HSPCs (D) and HSCs (E) in vehicle (Veh)- or Nec1s-treated mock- or infected WT mice 7 d.p.i. n = 4–5 mice/group. *P < 0.05, **P < 0.005, ***P < 0.001, ****P < 0.0001. **(E)** Survival of IOE-infected WT mice treated with Veh or Nec-1s, as depicted in (A), n = 8 mice/group. **(F)** Bacterial burden in the spleen of Veh- and Nec-1s-treated IOE-infected WT mice at 7 d.p.i., represented as *Dsb* copy/50ng total DNA.

To test the impact of RIPK1 signaling on IFNα/β-dependent HSPC depletion we next treated IOE-infected WT mice Nec-1s (Fig 7A). RIPK1 antagonism rescued HSPC numbers, reduced dead/dying HSPCs, and expanded the HSC pool above that of mock-infected controls (Fig 7B–7D). Blocking RIPK1 signaling using Nec-1s during acute IOE infection rescued HSPC and HSC depletion in IOE infection, though it did not improve survival (Fig 7E). The failure of Nec-1s to protect mice, despite the remarkable rescue of the hematopoietic compartment may be due to high bacterial burden in Nec-1s treated mice, which was similar to vehicle-treated mice (Fig 7F).

### Necrostatin-1s treatment improves hematopoietic recovery from shock-like infection

**Fig 8:**
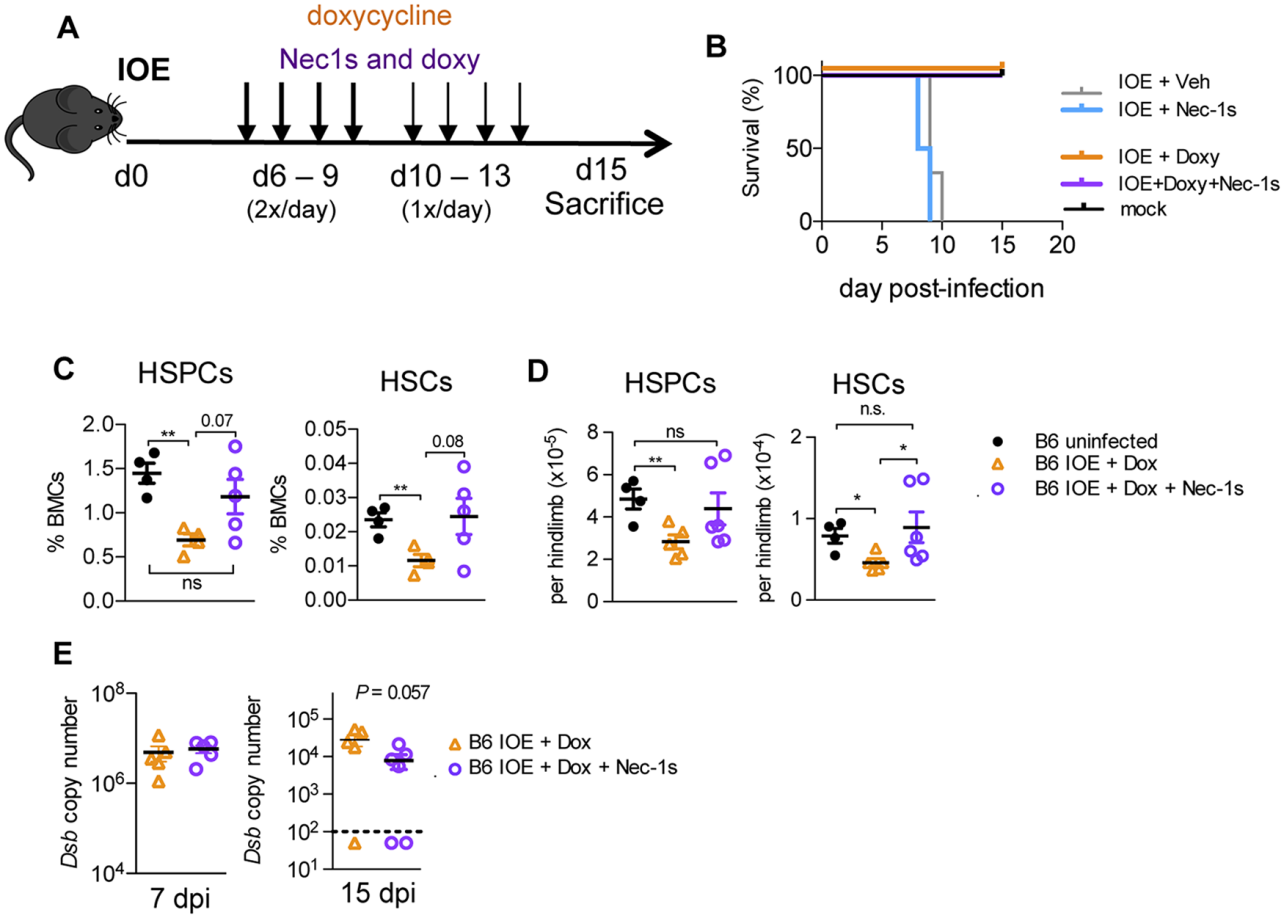
Targeting RIPK1 improves hematologic recovery in IOE infection survivors. (**A**) Mice were mock- or IOE-infected mice and treated with vehicle (Veh), Nec-1s, doxycycline (Doxy)-, or Doxy+ Nec-1s, as shown. (**B**) Survival of mock- (black line) or IOE-infected mice treated with vehicle (gray line), Nec1s (blue line), Doxy (orange line), or Doxy + Nec1s (purple line). (**C**) Surviving mice were evaluated at 15 d.p.i. for frequencies of BM HSPCs and HSCs, n = 5 mice/group, 4 mice/mock group. (**D**) Numbers of HSPCs and HSCs in mock and Veh-, Doxy-, and Doxy + Nec-1s-treated mice 15 d.p.i (**E**) Bacterial burden in the spleens of Doxy-, and Doxy + Nec-1s-treated mice 7 and 15 d.p.i., represented as *Dsb* copy/50ng total DNA. n = 3–5 mice/group. Dashed line represents the limit of detection. * *P* < 0.05, ** *P* < 0.001.

Early antibiotic treatment with doxycycline (doxy) is the current standard of care for patients with human monocytic ehrlichiosis, and related rickettsial infections, as delayed diagnosis is common and associated with poor outcomes [31]. Severe immune-mediated pathology can occur independently of bacterial burden [56], however, and survivors of sepsis-induced shock suffer long-term immunosuppression and other sequelae [57]. To control bacterial growth and evaluate the impact of RIPK1 on hematologic recovery from shock-like infection, we administered antibiotics (doxycycline; doxy) in conjunction with Nec-1s to WT IOE-infected mice. To mimic delayed diagnosis, common in rickettsial infections [31], we began treatment on day 6 post-infection (Fig 8A). Without antibiotics, or with Nec1s alone, IOE-infected mice succumbed to infection by day 10, whereas mice administered doxy survived (Fig 8B), and could be subsequently analyzed. IOE-infected mice administered doxy alone exhibited significantly reduced frequencies and numbers of HSPCs and HSCs at 15 d.p.i., relative to mock-infected control mice (Fig 8C and 8D). However, co-administration of Nec-1s with doxy resulted in increased HSPC and HSC frequencies 15 d.p.i., resembling those seen in uninfected mice. Furthermore, treatment with both doxy + Nec-1s resulted in HSPC numbers similar to those in mock-infected mice and a significant increase in HSCs relative to mice treated with doxy alone. Bacterial burden was similar during acute infection (7 d.p.i.) between treatment groups, however, during the recovery phase (15 d.p.i.), bacterial burden was lower in mice that received Nec-1s in addition to doxy (Fig 7E). These data demonstrate that RIPK1 activity impairs hematopoiesis during acute infection, and has lasting effects on HSC and HSPC numbers. Our findings suggest that RIPK1 activity can protract disease and RIPK1 antagonism may be therapeutically relevant for improving hematopoiesis and long-term outcomes in patients surviving acute, severe infections where type I IFNs are elevated.

## Discussion

Demand-adapted hematopoiesis is a dynamic process sculpted by cytokines, and can vary depending upon the duration and magnitude of stimulation and the presence of secondary stimuli [1, 4, 58]. IOE infection causes lethal, shock-like disease accompanied by cytopenias and immune dysfunction, similar to what is observed in patients with Rocky Mountain spotted fever and severe hemorrhagic viral infections. IOE is unusual for the ehrlichia because it infects endothelial cells [59], and it elicits shock-like infection driven by TNFα and type I IFNs [20, 28, 29]. We previously found that blocking type I IFN signaling in both the hematopoietic and non-hematopoietic system is necessary for protection against lethal infection [29], and herein we demonstrate a profound impact of type I IFNs on hematopoiesis and HSC and HSPC function. Hematopoiesis produces mature immune cells that can impact immunity and host defense [3, 60], thus the impact of infection and type I IFNs on HSPCs is directly relevant to recovery and survival in patients.

**Fig 9:**
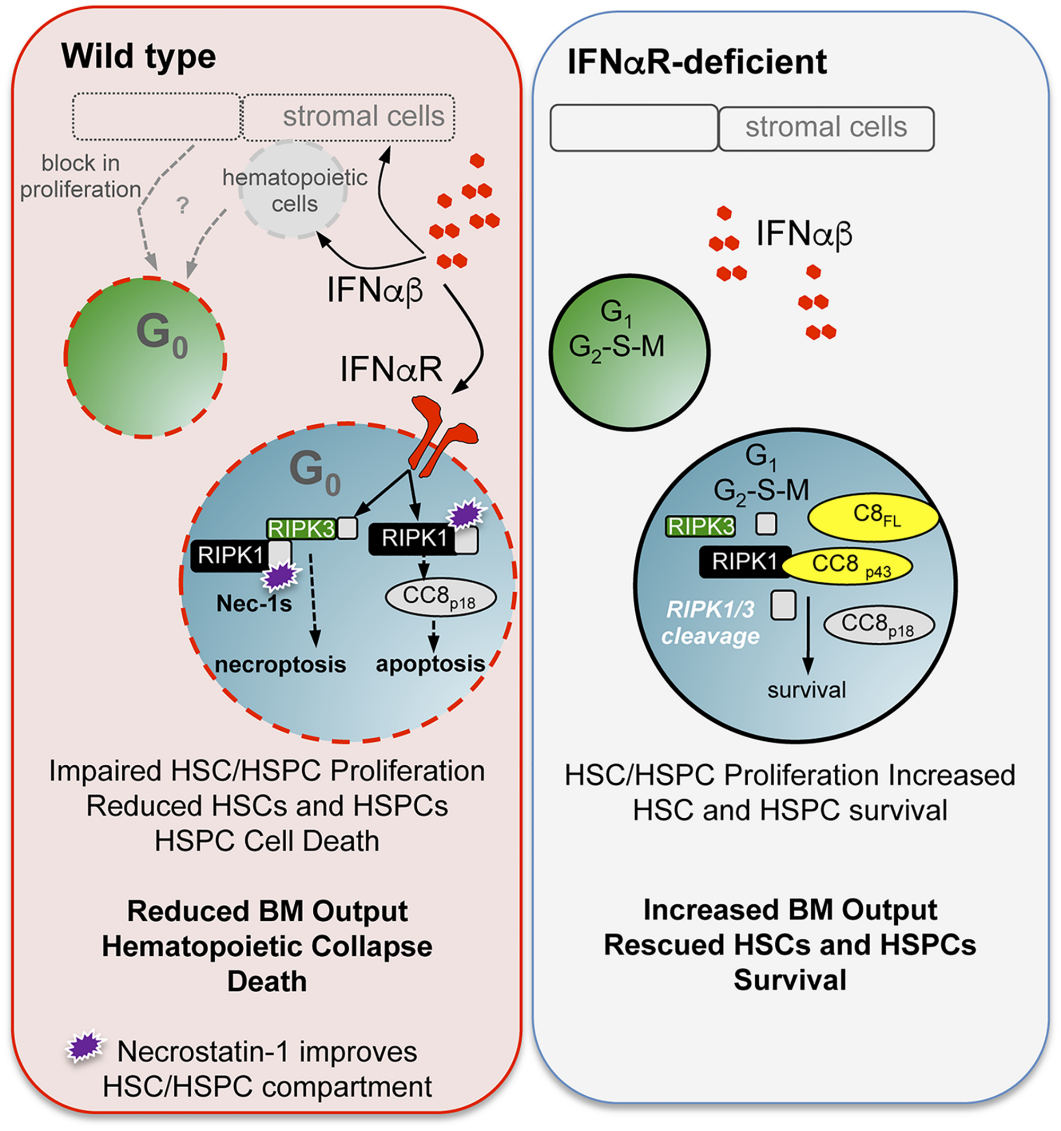
Summary model. *Ixodes ovatus* ehrlichia results in lethal shock-like infection and hematopoietic collapse. During infection of WT mice (left panel), type I IFN levels were elevated in the bone marrow, inducing hematopoietic stem cell (HSC) and progenitor (HSPC) loss. Type I IFNs restricted entry into the cell cycle, and type I IFN receptor (IFNαR) stimulation induced HSPC cell death. IFNαR signals limited caspase 8 expression and activation, blocked RIPK1 and RIPK3 cleavage, and caused RIPK1-kinase dependent cell death. In the absence of IFNαR signaling (right panel), HSCs and HSPCs were more proliferative and exhibited improved survival. IFNαR-deficiency results in increased pro-caspase 8 (C8_FL_), cleaved caspase products (C8_p43_ and C8_p18_), and increased cleavage of RIPK1 and RIPK3, resulting in improved HSC/HSPC numbers and function.

In sterile inflammation, type I IFNs drive proliferation and apoptosis of HSPCs and HSCs [8, 39, 40]. Based on our previous observation that IFNα/β promotes lethal disease in shock-like infection [29], we predicted the impact of IFNα/β on HSCs may differ between sterile and infection-dependent inflammation. A key finding from our studies is that in contrast to sterile inflammation, type I IFNs drive hematopoietic dysfunction via impaired proliferation and increased RIPK1-dependent cell death that may include both RIPK1-dependent apoptosis and necroptosis (Fig 9). Whereas IFNα/β directly reduced HSPCs, stem cell numbers were not directly responsive to IFNα/β. Rather, type I IFNs were indirectly associated with HSC loss and increased quiescence during infection. While HSC dormancy may be critical for preserving the stem cell population under inflammatory or infectious conditions, failure to proliferate in response to infectious demand was associated with impaired myelopoiesis and reduced host innate immunity. Additional studies are warranted to determine the IFNα/β dependent factors associated with impaired proliferation.

Similar to sterile inflammation, infection increased progenitor cell death. However, during infection type I IFNs mediated HSPC death by reducing caspase 8 expression and activity, which correlated with reduced RIPK1 and RIPK3 cleavage in the BM. Our conclusion that type I IFNs induce RIPK1-dependent apoptosis and/or necroptosis is based on the specific protection of HSPCs conferred by RIPK1 antagonism, the intrinsic role for RIPK3 kinase activity in HSPC non-apoptotic cell death, and the observation of limited caspase 8 expression and activity in WT mice. Our findings do not exclude a role for RIPK1-dependent apoptosis, which is also kinase-dependent and therefore sensitive Nec-1s [61]. WT mice exhibit profound rescue of HSCs and HSPCs upon RIPK1 antagonism with Nec-1s, similar to what is observed in *Ifnar1*^*-/-*^-deficient mice, consistent with a central role for RIPK1 kinase activity in the demise of the hematopoietic compartment in IOE infection.

IFNαR signaling potentiates necroptotic RIPK3 signaling and primes TNFR2 and MLKL expression in macrophages [44, 55]. Reconstitution of *Ifnar1*^*-/-*^ macrophages with TNFR2 and MLKL did not fully restore necroptotic sensitivity [44], suggesting that additional IFNαR-dependent signals regulate necroptosis. Neither *Ifnar1*^-/-^ whole BM nor purified HSPCs were deficient in RIPK3 or MLKL expression. Caspase 8 enzymatic activity negatively regulates necroptosis via RIPK1 and RIPK3 instability [42], and we found that type I IFNs limited caspase expression during infection, providing a potential mechanism whereby IFNα/β facilitate pathologic RIPK1-RIPK3 interaction and downstream RIPK3 activation.

Caspase 8 performs numerous and complex roles in regulating cell death and survival pathways. Caspase 8 is a critical initiator of the extrinsic pathway of apoptosis, as it cleaves effector caspases, including caspase 3, but can limit necroptosis by impairing RIPK1 and RIPK3 interactions. Caspase 8 can also form a complex with RIPK3 to activate inflammasome-independent IL-1β production [62], which may underlie the increase in IL-1β observed in the absence of IFNαR. The IFNα/β-dependent reduction in caspase activity during IOE infection is distinct from the impact of sterile inflammation on HSPCs, where increased cleaved caspase 3 was observed [8]. Despite increased caspase 3/7 activity in infected *Ifnar1*^-/-^ mice we failed to detect increased apoptosis in IOE-infected *Ifnar1*^-/-^ mice. The striking increase in full length pro-caspase 8, as well as active caspase 8, in *Ifnar1*^-/-^ mice was apparent prior to infection, suggesting *Ifnar1*^-/-^ mice may be poised to resist RIPK1-dependent cell death. Caspase 8 activity is also associated with proliferation in the hematopoietic compartment [48, 63], and in lymphocytes wherein caspase-8-dependent cleavage induces more efficient recruitment of RIPK1, necessary to activate NF-kB [64]. An intriguing possibility is that increased survival and proliferation of HSCs and HSPCs in *Ifnar1*^-/-^ mice is dependent on elevated caspase 8 activity consistent with our observation that the pool of HSCs expands during IOE-infection in *Ifnar1*^-/-^ mice, and the extreme sensitivity of HSCs and HSPCs to zVAD.

How type I IFNs regulate caspase 8 during infection is an important and open question. Caspase 8 can be degraded by autophagy [65], a pro-survival strategy to counter cellular stress and infection, and, of note, type I IFNs can initiate autophagy [66]. Interestingly, the *Ehrlichia* can also induce autophagy, and subsequently seize nutrients for their growth, and inhibition of autophagy controls ehrlichial growth [67]. As reduced bacterial burden is observed in *Ifnar1*^-/-^ mice, our data are consistent with a potential role for IFNαR-dependent signaling in promoting autophagy which would support bacterial growth and, at the same time, reduce caspase 8, licensing RIPK1-dependent cell death. However, caspase 8 can also blunt autophagy by degrading components of the autophagic macherinery [68], and therefore elevated caspase 8 in *Ifnar1*^-/-^ mice may prevent autophagy, thus limiting bacterial growth. Additional studies to interrogate the precise mechanisms of caspase 8 regulation may reveal important regulatory nodes critical for the control of bacterial growth and cell survival.

RIPK1 and RIPK3 can promote inflammasome activity, however, *caspase-1* and *Nlrp3*-deficient mice were not protected from IOE infection [28]. IFNαR- and TLR9-mediated caspase-11 activation has recently been observed during IOE infection [34], and caspase-11 activity can promote pyroptotic cell death by cleaving gasdermin D [69]. Due to the robust improvement of HSPC and HSC numbers upon RIPK1 antagonism, we reason that caspase 11-mediated pyroptosis is unlikely to cause loss of HSCs and HSPCs, though pyroptotic cell death may be important for pathology in other tissues during disease, such as the liver and lung, two organs that are heavily infected [70]. In line with this possibility, Nec-1s-treated mice died with similar kinetics as vehicle treated, thus, caspase 11 activity in non-hematopoeitic cells may contribute to pathology and death during IOE infection.

Determining the *in vivo* relevance of various cell death pathways is challenging for a number of reasons. Depleting or blocking signaling components can render cells more susceptible to other death pathways [71], which complicates interpretations of *in vivo* observations. RIPK3 is a key effector in necroptotic cell death, but can also drive apoptosis or pyroptosis and regulate cytokine production. In addition, the distinct cellular context, with respect to signaling components and negative regulators of necroptosis, may regulate cell type-specific differences in cell death during infection. It is also important to consider that certain cell death pathways may be critical for control of infection [72]. Indeed, RIPK1-dependent apoptotic cell death is necessary for control of *Yersinia pestis* [73]. Particularly challenging is addressing cell death processes *in vivo*, as distinct cell types respond uniquely to stimuli, and may influence other cell types. As HSCs generate all mature cells, it is difficult to parse out the intrinsic pathways that govern cell death and survival. Using mixed BM chimeric mice we were able to define an intrinsic role for type IFNs and RIPK3 in the loss of HSPCs. Despite these complexities, delineating the functional consequences of inflammatory cell death pathways will improve the development of therapeutics to treat infection-induced hematopoietic dysfunction and suppression.

Our work highlights the potential utility of targeting RIPK1 to mitigate BM failure in patients exposed to high levels of IFNα/β. Nec-1s was not able to protect mice against death, likely due to the failure of Nec1s alone to control bacterial growth and additional type I IFN-dependent effects on non-hematopoietic tissues. However, addition of Nec-1s to antibiotic treatment improved HSC and HSPC numbers, which may prove clinically relevant. Necroptosis-promoting cytokines, including IFNα/β and TNFα, are induced by numerous disease states, including infection, as well as clinical protocols, such as radiation and BM transplantation [74, 75], and are a potential prognostic biomarker in patients with sepsis [76]. Therefore, therapeutic targeting of type I IFNs and/or RIPK1 and RIPK3 may prevent hematologic dysfunction in such scenarios.

## Materials and methods

### Mice and ethics statement

C57BL/6 mice (CD45.2) and a CD45 congenic strain (B6.SJL-*Ptprc*^*a*^/Boy-AiTac; CD45.1) were purchased from Taconic (Petersburgh, NY). IFNα/β receptor gene (*Ifnar1*)-knockout, C57BL/6J-*Ripk3*^*m1Btlr*^/J(*Ripk3*^Δintron2^; JAX stock # 025738), β-Actin (β-act) enhanced green fluorescent protein (EGFP) [B6-Tg(CAG-EGFP)131Osb/LeySopJ] mice expressing EGFP, driven by the β-act promoter, and C57BL/6-TG(UBC-GFP)30Scha/J mice expressing GFP, were purchased from Jackson Laboratory (Bar Harbor, ME). *Tnfrsf1a*^-/-^ *Tnfrsf1b*^-/-^ (TNFRI and II double knockout mice) mice were a kind gift of Dr. Lei Jin (University of Florida). All other mice were bred in the Animal Resources Facility at Albany Medical College under specific-pathogen-free conditions. Male and female mice between 6–12 weeks of age were used for experiments. All experiments were approved by Albany Medical College’s Animal Care and Use Committee.

### Bacteria

*Ixodes ovatus* ehrlichia (IOE) bacteria were obtained from infected mouse splenocytes, as previously described [77]. 6–8 week old mice were infected, via intraperitoneal injection, with 300,000 to 1x10^6^ IOE bacteria in sucrose-phosphate-glutamate (SPG buffer). Mock-infected mice were injected intraperitoneally (I.P.) with equivalent volumes SPG buffer.

### Sterile immune stimulation

Endotoxin-free Polyinosinic:polycytidylic acid (PolyI:C; InvivoGen) was administered by intraperitoneal injection at 10mg/kg in saline at days 0, 2, 4, and 6.

### Antibody neutralization

Anti-IFNα (rat anti-IFNα [RMMA-1]) and anti-IFNβ (rabbit anti-IFNβ) antibodies were obtained from PBL Interferon Source and were administered to WT mice at days 4–7 post-infection, as previously described [29].

### Quantification of bone marrow cell types

Bone marrow was liberated from femora, tibiae, or pelves by flushing or crushing and filtering through a cell strainer. Single-cell, red blood cell-depleted suspensions were plated, washed and stained with appropriate antibodies. Data was acquired on an LSRII flow cytometer (BD Biosciences). Phenotypic definitions were as follows, unless otherwise noted: Hematopoietic stem cell (HSC; CD150^+^ CD48^-^ CD135^-^ c-Kit^+^ Lineage^-^), Stem and progenitor cell pool (HSPC; c-Kit^+^ Lineage^-^), Common myeloid progenitor (CMP; CD34^+^ CD16/32^-^ CD127^-^ c-Kit^+^ Lineage^-^), Granulocyte macrophage progenitor (GMP; CD34^+^ CD16/32^+^ CD127^-^ c-Kit^+^ Lineage^-^), monocyte (CD11b^+^ Ly6C^hi^).

### Isolation of hematopoietic stem and progenitor cells

HSC or HSPC populations were sort-purified using a FACSARIA II (BD Biosciences) into media or directly onto poly-L-lysine-coated 12mm coverslips.

### Transplantation

For analysis of hematopoietic stem and progenitor cell function, recipient mice were lethally irradiated (950 rad, administered in 2 doses, 4 hours apart) and administered HSCs or HSPCs via retroorbital injection. Mice also received 3 x 10^5^ protective whole bone marrow cells. Blood was screened beginning at 4 weeks for up to 16 weeks. For generation of mixed bone marrow chimeric mice, CD45 congenic mice were lethally irradiated (950 rad, administered in 2 doses, 4 hours apart). Irradiated mice received a total of 10x10^6^ BM cells derived from WT CD45.1-expressing mice (B6.SJL-*Ptprc*^*a*^/Boy-AiTac) and *Ifnar1*-deficient CD45.2-expressing mice, or from C57BL/6-TG(UBC-GFP)30Scha/J mice (UBC-GFP) and *Ripk3*^*Δintron2*^ (C57BL/6J-*Ripk3*^*m1Btlr*^/J (JAX stock # 025738)) mice at a 1:1 ratio.

### Analysis of cell death

Cellular viability was determined using 7-aminoactinomycin D or Zombie fixable viability dye (BioLegend). Caspase activity was assayed by incubating freshly isolated bone marrow cells with FAM-LETD-FMK caspase 8 and FAM-DEVD-FMK caspase 3/7 fluorescent inhibitor probes (ImmunoChemistry Technologies) according to manufacturer’s instructions. Caspase activity was analyzed within viable cell populations.

### ELISA

BM cell lysates for protein analysis were homogenized manually with a pestle in a buffer containing IGEPAL CA-630 and proteinase inhibitors. Total protein concentration was measured using a BCA kit (Pierce) and a 23-Plex Inflammatory Cytokine Kit (Bio-Rad). ELISA kits for IFNα and IFNβ protein were purchased from eBioscience and PBL Interferon, respectively. Protein levels were normalized to total BM protein (mg).

### Western blot analysis

Western blot analysis was performed in whole bone marrow cell and sort-purified HSPC lysates. Antibodies used were RIPK3 (ProSci), MLKL (3H1; EMD Millipore), caspase 8 (R & D systems), RIPK1 (BD Transduction Labs) actin (BD Transduction Labs), and cyclophilin B (Cell Signaling Technology). For analysis of MLKL in HSPCs, protein samples were concentrated using the Chemicon Protein-Concentrate kit (EMD Millipore).

### Small molecule administration

Mice received 5mg/kg zVAD-FMK (InvivoGen) I.P. once per day on days 4 and 6 post-IOE infection or on days 4, 5, and 6 post-infection. Mice received 100 μg Necrostatin-1s (Nec-1s; BioVision) by I.P. injection twice per day 3–6 days post-infection, or twice per day 6–9 days post-infection and once per day 10–13 days post-infection. Mice received 10 mg/kg doxycycline by I.P. injection once or twice per day 6–9 days post-infection, and once per day 10–13 days post-infection.

### Immunofluorescence staining

Hematopoietic stem and progenitor cells were fixed for 10 minutes at room temperature in a 2% formaldehyde solution containing 9.8 mM NaIO_4_, and 62 mM lysine after 5 minutes of adherence to coverslips. Cells were permeabilized for 15 minutes in 0.02% saponin at room temperature and then incubated in mouse anti-RIP1 [38/RIP] from BD Biosciences and rabbit anti-RIP3 from ProSci, Inc. (clones that were previously published [55]) for 1 hour. Species-specific, fluorescently-conjugated IgG (H+L) F(ab’)_2_ fragments from Life Technologies or Cell Signaling Technology were used for detection. Coverslips were mounted with Electron Microscopy Sciences Fluorogel II containing DAPI.

### Confocal microscopy and image analysis

Images were acquired on a Zeiss LSM510 META-NLO laser-scanning microscope system equipped with a Plan-Apochromat x63/1.4 numerical aperture oil objective lens, an argon laser (at 488 nm), a green HeNe diode laser (at 543 nm), and standard emission filters for Alexa488 and 555. Analysis of RIPK1 and RIPK3 signal association was performed using the colocalization module of Imaris (BitPlane, Oxford Instruments). Briefly, a colocalization channel was generated and used to calculate the Pearson’s coefficient of the colocalized volume.

### Cell cycle analysis

Cell cycle status was analyzed by intracellular staining for Ki-67 (M-19; Santa Cruz Biotechnology Inc.), and 4’,6-diamidino-2-phenylindole (DAPI) was added 15 minutes prior to analysis.

### Quantification and statistical analysis

#### Flow cytometry

Flow cytometry data were analyzed using FlowJo software (TreeStar) using appropriate fluorescence minus one controls.

#### Densitometry

Band intensity was measured from original digital immunoblot images using Image Lab software (Bio-Rad). Briefly, band intensity of protein of interest was divided by actin band intensity for each sample and normalized values were graphed. For full length and cleaved caspase 8 analysis, the normalized cleaved caspase 8 (p18) band intensity was divided by the normalized full length caspase 8 band intensity.

#### Quantification of bacterial burden

50 ng DNA was extracted from splenocyte lysates using the DNeasy Blood and Tissue kit (Qiagen), and used to quantify disulfide bond formation protein (*Dsb*) gene by real-time quantitative PCR, as previously described [29]. Mice with fewer than 10^3^ *Dsb* copies/50ng DNA were excluded, as this is the limit of detection.

#### Statistical analysis

Data was analyzed using GraphPad Prism, version 5. Statistical analyses of data sets including multiple time points or >2 experimental groups were performed using two- or one-way ANOVA with Tukey multiple comparison post-tests. Statistical analyses of two experimental groups at a single time point were performed using two-tailed Student’s t-test. All data are represented as mean ± standard error of the mean, and are representative of at least two independent experiments, unless otherwise noted.

## Supporting information

S1 Fig Type I IFNs suppress myelopoeisis and extramedullary hematopoiesis during IOE infection. **(A)** BM and spleen cells were isolated and 2X10^4^ BM cells or 2X10^5^ splenic cells were seeded into each 35 mm-dish containing 1 ml of methocult media. Cells were incubated for 7 days, and thereafter colony-forming units were counted under a microscope. Myeloid colony formation of indicated colony forming unit (CFU) in BM cells isolated 7 days post IOE infection (d.p.i.). GEMM: granulocyte, erythrocyte, monocyte, megakaryocyte; GM: granulocyte, monocyte; G: granulocyte; M: monocyte; E: erythrocyte. n = 3–4 mice/group. **(B)** Differentiation of Lin^-^ BM cells harvested 7 d.p.i. Briefly, 500 Lin- BM cells or 1000 Lin-spleen cells were seeded into each well of a 24-well plate containing irradiation (3000 rads) treated OP-9 cells in the presence of IL-3, IL-7, GM-CSF, SCF and Flt3L, and cultured for 10 days. Cells were analyzed to identify monocytes (CD11b+ Ly6C^hi^ Ly6G^-^) and neutrophils (CD11b+ Ly6C^-^ Ly6G^+^). n = 5–7 mice/group. *P < 0.05, **P < 0.001, ***P < 0.0001. **(C)** Flow cytometry plots of WT and *Ifnar1*^-/-^ singlet peripheral blood cells to evaluate circulating Lin^-^ c-Kit^+^ -defined HSPCs in mock and 6, 7, and 9 d.p.i. **(D)** Splenic cellularity throughout IOE infection. n = 3–13 mice/group. **(E)** Phenotypic HSCs in the spleen. n = 3–11 mice/group. *P* < 0.0001 for *Ifnar1*^-/-^ vs. WT (in D and E). **(F)** Myeloid colony formation of indicated CFU among splenocytes harvested 7 d.p.i. n = 3–4 mice/group. *P < 0.01, **P < 0.0001. **(G)** Differentiation of Lin^-^ splenocytes harvested 7 d.p.i. and cultured for 10 days on OP-9 stromal cells, 500 Lin^-^ cells per well. n = 5–7 mice/group. *P < 0.01. **(H-I)** Monocytes as analyzed by flow cytometry (CD11b+ Ly6C^hi^ Ly6G^-^) in the bone marrow and spleen. n = 3–13 mice/group. **(J-K)** Neutrophils as analyzed by flow cytometry (CD11b+ Ly6C^-^ Ly6G^+^) in the bone marrow and spleen throughout IOE infection. n = 3–13 mice/group.
(TIFF)

S2 Fig IOE-induced IFNα/β impair the multilineage hematopoietic reconstituting activity of HSCs. **(A)** Reconstitution of indicated hematopoietic lineages in the blood, 16 weeks post-primary transplant of WT and *Ifnar1*^-/-^ Lin^-^ c-Kit^+^ (LK)-defined HSPCs at steady-state. n = 7 recipients. **(B)** Reconstitution of indicated lineages in the peripheral blood, 16 weeks post-primary transplant of HSCs derived from IOE-infected mice. n = 6 recipients.
(TIFF)

S3 Fig IOE-induced IFNα/β modulate cytokine and chemokine production in the BM. **(A-G)** Inflammatory cytokines and chemokines in the BM of WT and *Ifnar1*^-/-^ mice at indicated days post-infection (d.p.i.). n = 3–9 mice/group.
(TIFF)

S4 Fig IFNαR-dependent HSC and HSPC depletion during sterile inflammation is associated with caspase activity and cell death. **(A)** Schematic depicting repeated 10mg/kg polyI:C (pI:C) stimulation of WT and *Ifnar1*^-/-^ mice. **(B)** BM cellularity of WT and *Ifnar1*^-/-^ mice harvested at indicated time points, collected 24 hours after the preceding pI:C stimulation. n = 4–6 mice/group. *P* < 0.02 for WT vs. *Ifnar1*^*-/-*^. **(C-D)** HSCs and HSPCs per hindlimb of pI:C-stimulated WT and *Ifnar1*^-/-^ mice at indicated time points. n = 4–6 mice/group. (**E-F**) Proportion of dead/dying Lin^-^ CD48^-^ BM cells (**E**) and proportion of live Lin^-^ CD48^-^ BMCs with caspase 8 and/or caspase 3/7 activity (**F**) in pI:C-stimulated WT and *Ifnar1*^-/-^ mice. n = 4–6 mice/group.
(TIFF)

S5 Fig Cell death during IOE infection. **(A)** Representative flow cytometry plots demonstrating Annexin V and 7AAD staining of HSPCs in mock and IOE- infected WT and *Ifnar1*^-/-^ mice 6 d.p.i. **(B)** Fold change in populations of apoptotic and dead cells on days 6 and 8 post-infection in WT and *Ifnar1*^-/-^ mice is shown, relative to mock-infected mice. Changes were not significant. n = 3–6 mice/group.
(TIFF)

S6 Fig IFNαR-signaling does not regulate TNFα, TNFR2, MLKL, and RIPK3 during IOE infection. **(A)** Levels of TNFα in the BM of WT and *Ifnar1*^-/-^ IOE-infected mice and indicated days post-infection (d.p.i.). n = 3–6 mice/group. **(B)** Histograms depicting CD120b (TNFR2) staining in lineage^-^ cKit^+^ (LK)-defined HSPCs from mock- and IOE-infected *Tnfr1/2*^*-/-*^, WT, and *Ifnar1*^*-/-*^ mice 7 d.p.i. **(C)** Immunoblot detection of RIPK3, MLKL, and cyclophilin B in BM cell lysates from 7 day IOE-infected WT and *Ifnar1*^*-/-*^ mice. n = 4 mice/group. **(D)** Immunoblot detection of total RIPK3 and MLKL from sort-purified WT and *Ifnar1*^*-/-*^ HSPCs at 7 d.p.i. n = 3 mice/group **(E-F)** Immunoblot detection of FADD and actin in BM cell lysates of WT and *Ifnar1*^-/-^ 7 day IOE-infected mice and a representative mock-infected control mouse. n = 3 mice/group.
(TIFF)

S7 Fig IFNαR-dependent increase in RIPK1 and 3 association in HSPCs during IOE infection. **(A)** Representative micrographs of RIPK3, RIPK1, and DAPI staining in HSPCs sorted from IOE-infected WT and *Ifnar1*^-/-^ mice 7 d.p.i. Scale bar = 1μm. **(B)** Pearson’s coefficient of RIPK1 and RIPK3 colocalization. n = 15–16 cells analyzed/group.
(TIFF)

S8 Fig RIPK3-kinase independent type I IFN production and HSPC death in IOE-infected mice. **(A)** IFNα and IFNb production in BM during IOE infection in WT and *Ripk3*^Δintron2^ mice at the indicated times post-infection. n = 3–4 mice/group. (**B**) RIPK3 detection in BM cell lysates from WT and *Ripk3*^Δintron2^ mice. (**C**) HSPC cell death (Annexin V and 7AAD staining) in WT and *Ripk3*^Δintron2^ mice on day 7 post-IOE infection. (**D**) *Ripk3*^Δintron2^ mice infected with IOE were treated with Nec-1s. Absolute frequencies of HSPCs and HSCs are shown in Veh-treated and Nec-1s treated mice.
(TIFF)
