# Supplementary figures and images for: Type I IFNs drive hematopoietic stem and progenitor cell collapse via impaired proliferation and increased RIPK1-dependent cell death during shock-like ehrlichial infection
Source: PLoS Pathog. 2018 Aug 6;14(8):e1007234. doi: 10.1371/journal.ppat.1007234 (PMC6095620; doi:10.1371/journal.ppat.1007234)

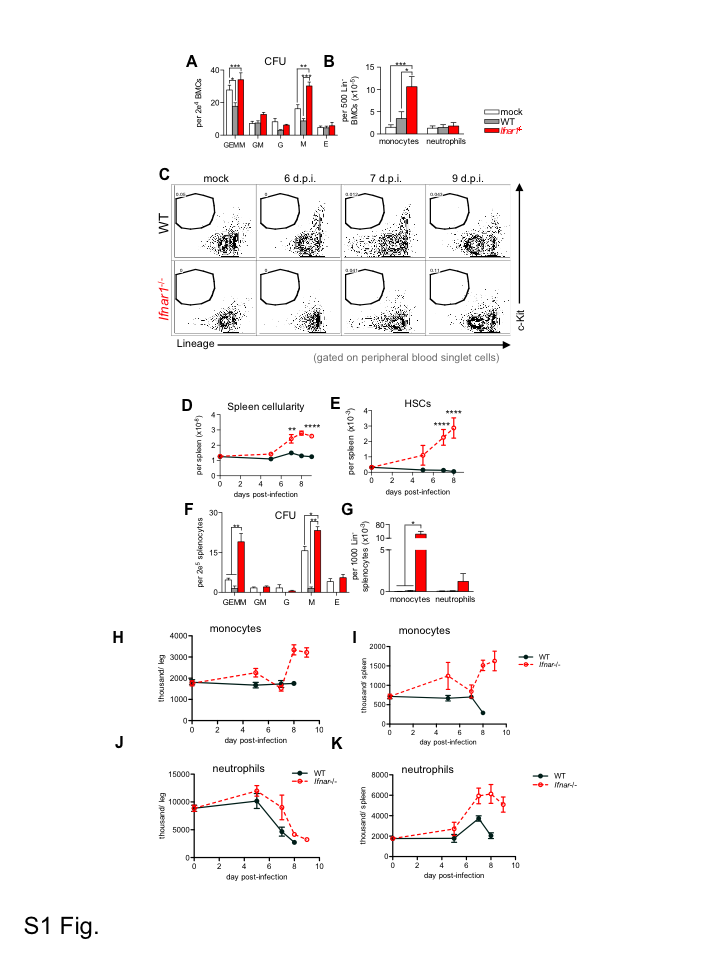

Supplement: S1 Fig — (A) BM and spleen cells were isolated and 2X104 BM cells or 2X105 splenic cells were seeded into each 35 mm-dish containing 1 ml of methocult media. Cells were incubated for 7 days, and thereafter colony-forming units were counted under a microscope. Myeloid colony formation of indicated colony forming unit (CFU) in BM cells isolated 7 days post IOE infection (d.p.i.). GEMM: granulocyte, erythrocyte, monocyte, megakaryocyte; GM: granulocyte, monocyte; G: granulocyte; M: monocyte; E: erythrocyte. n = 3–4 mice/group. (B) Differentiation of Lin- BM cells harvested 7 d.p.i. Briefly, 500 Lin- BM cells or 1000 Lin-spleen cells were seeded into each well of a 24-well plate containing irradiation (3000 rads) treated OP-9 cells in the presence of IL-3, IL-7, GM-CSF, SCF and Flt3L, and cultured for 10 days. Cells were analyzed to identify monocytes (CD11b+ Ly6Chi Ly6G-) and neutrophils (CD11b+ Ly6C- Ly6G+). n = 5–7 mice/group. *P < 0.05, **P < 0.001, ***P < 0.0001. (C) Flow cytometry plots of WT and Ifnar1-/- singlet peripheral blood cells to evaluate circulating Lin- c-Kit+ -defined HSPCs in mock and 6, 7, and 9 d.p.i. (D) Splenic cellularity throughout IOE infection. n = 3–13 mice/group. (E) Phenotypic HSCs in the spleen. n = 3–11 mice/group. P < 0.0001 for Ifnar1-/- vs. WT (in D and E). (F) Myeloid colony formation of indicated CFU among splenocytes harvested 7 d.p.i. n = 3–4 mice/group. *P < 0.01, **P < 0.0001. (G) Differentiation of Lin- splenocytes harvested 7 d.p.i. and cultured for 10 days on OP-9 stromal cells, 500 Lin- cells per well. n = 5–7 mice/group. *P < 0.01. (H-I) Monocytes as analyzed by flow cytometry (CD11b+ Ly6Chi Ly6G-) in the bone marrow and spleen. n = 3–13 mice/group. (J-K) Neutrophils as analyzed by flow cytometry (CD11b+ Ly6C- Ly6G+) in the bone marrow and spleen throughout IOE infection. n = 3–13 mice/group. (TIFF) [file ppat.1007234.s001.tiff]

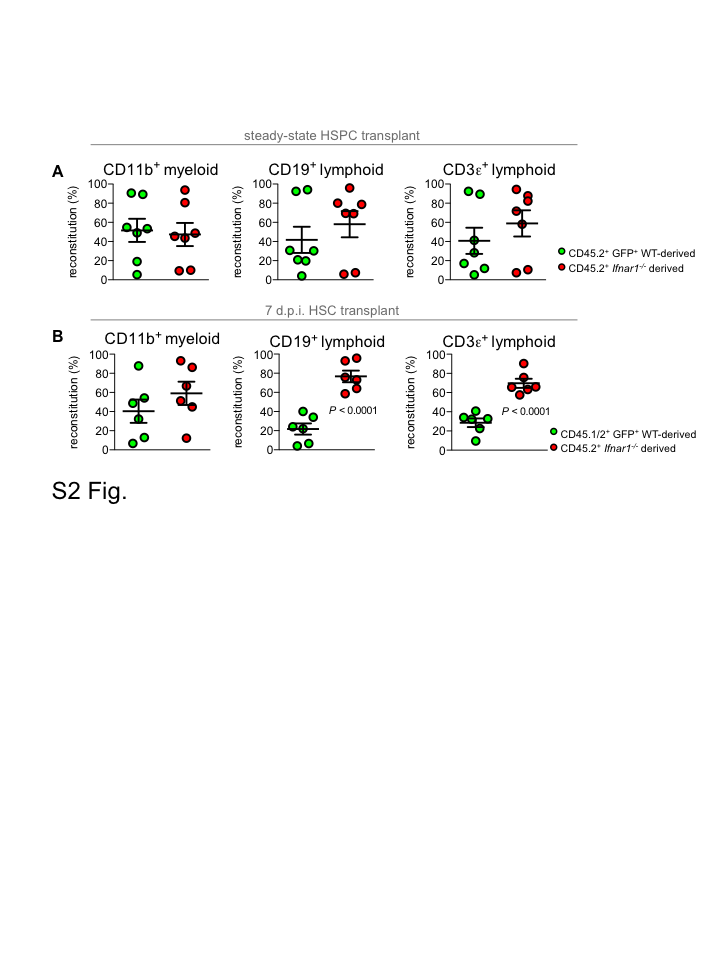

Supplement: S2 Fig — (A) Reconstitution of indicated hematopoietic lineages in the blood, 16 weeks post-primary transplant of WT and Ifnar1-/- Lin- c-Kit+ (LK)-defined HSPCs at steady-state. n = 7 recipients. (B) Reconstitution of indicated lineages in the peripheral blood, 16 weeks post-primary transplant of HSCs derived from IOE-infected mice. n = 6 recipients. (TIFF) [file ppat.1007234.s002.tiff]

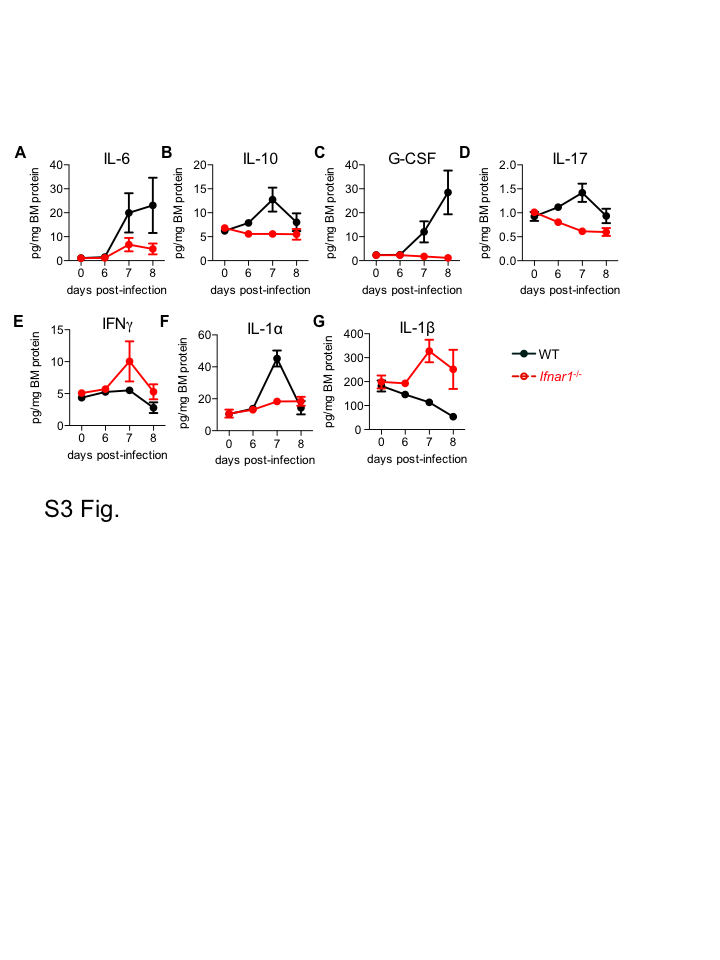

Supplement: S3 Fig — (A-G) Inflammatory cytokines and chemokines in the BM of WT and Ifnar1-/- mice at indicated days post-infection (d.p.i.). n = 3–9 mice/group. (TIFF) [file ppat.1007234.s003.tiff]

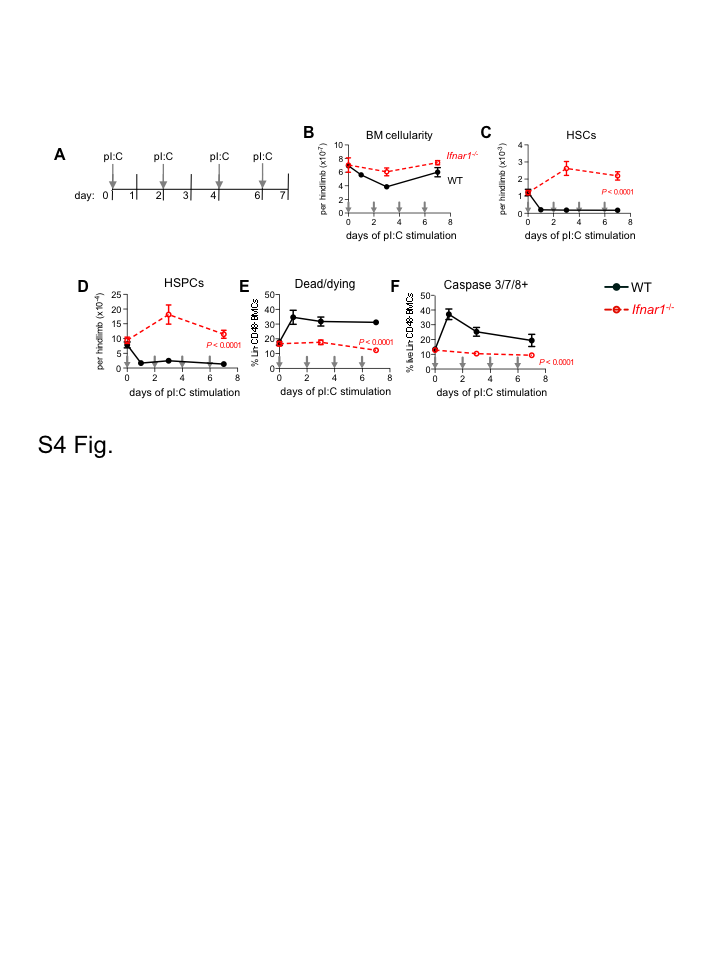

Supplement: S4 Fig — (A) Schematic depicting repeated 10mg/kg polyI:C (pI:C) stimulation of WT and Ifnar1-/- mice. (B) BM cellularity of WT and Ifnar1-/- mice harvested at indicated time points, collected 24 hours after the preceding pI:C stimulation. n = 4–6 mice/group. P < 0.02 for WT vs. Ifnar1-/-. (C-D) HSCs and HSPCs per hindlimb of pI:C-stimulated WT and Ifnar1-/- mice at indicated time points. n = 4–6 mice/group. (E-F) Proportion of dead/dying Lin- CD48- BM cells (E) and proportion of live Lin- CD48- BMCs with caspase 8 and/or caspase 3/7 activity (F) in pI:C-stimulated WT and Ifnar1-/- mice. n = 4–6 mice/group. (TIFF) [file ppat.1007234.s004.tiff]

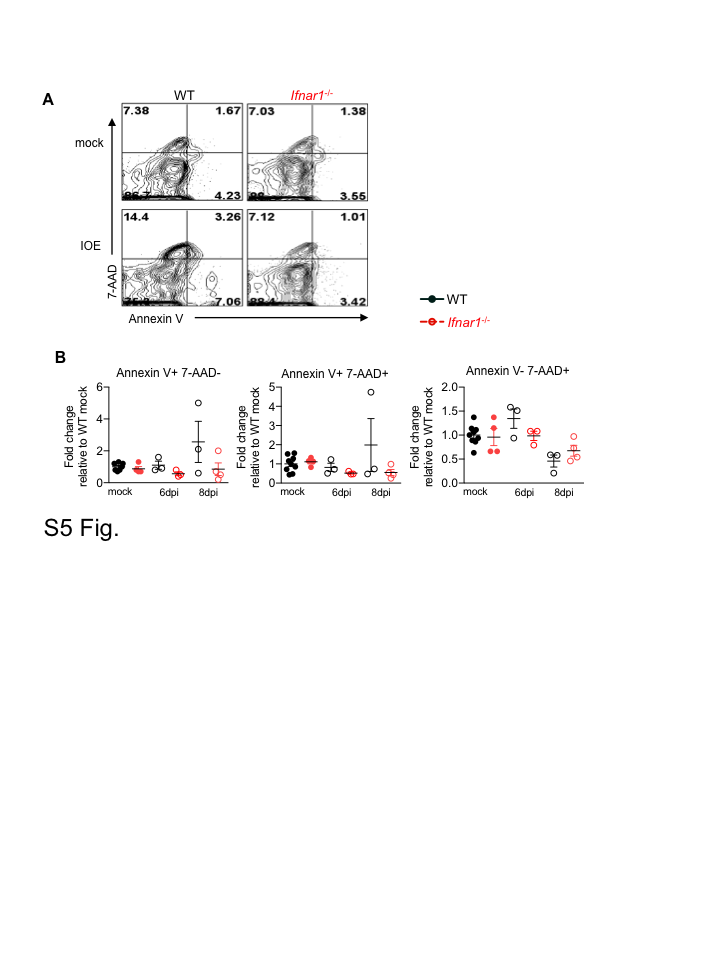

Supplement: S5 Fig — (A) Representative flow cytometry plots demonstrating Annexin V and 7AAD staining of HSPCs in mock and IOE- infected WT and Ifnar1-/- mice 6 d.p.i. (B) Fold change in populations of apoptotic and dead cells on days 6 and 8 post-infection in WT and Ifnar1-/- mice is shown, relative to mock-infected mice. Changes were not significant. n = 3–6 mice/group. (TIFF) [file ppat.1007234.s005.tiff]

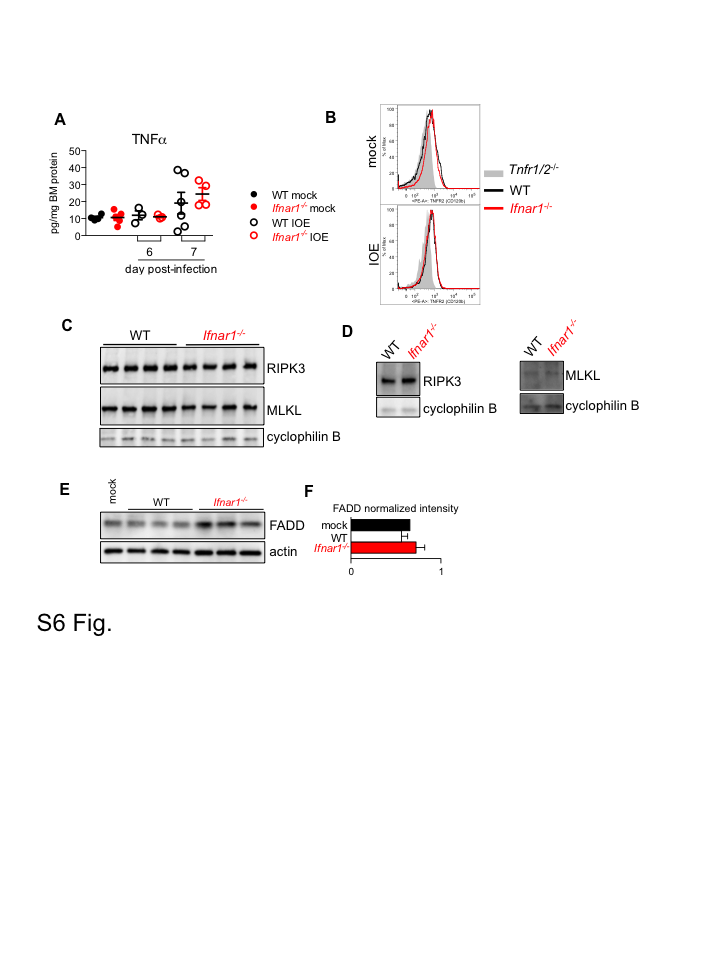

Supplement: S6 Fig — (A) Levels of TNFα in the BM of WT and Ifnar1-/- IOE-infected mice and indicated days post-infection (d.p.i.). n = 3–6 mice/group. (B) Histograms depicting CD120b (TNFR2) staining in lineage- cKit+ (LK)-defined HSPCs from mock- and IOE-infected Tnfr1/2-/-, WT, and Ifnar1-/- mice 7 d.p.i. (C) Immunoblot detection of RIPK3, MLKL, and cyclophilin B in BM cell lysates from 7 day IOE-infected WT and Ifnar1-/- mice. n = 4 mice/group. (D) Immunoblot detection of total RIPK3 and MLKL from sort-purified WT and Ifnar1-/- HSPCs at 7 d.p.i. n = 3 mice/group (E-F) Immunoblot detection of FADD and actin in BM cell lysates of WT and Ifnar1-/- 7 day IOE-infected mice and a representative mock-infected control mouse. n = 3 mice/group. (TIFF) [file ppat.1007234.s006.tiff]

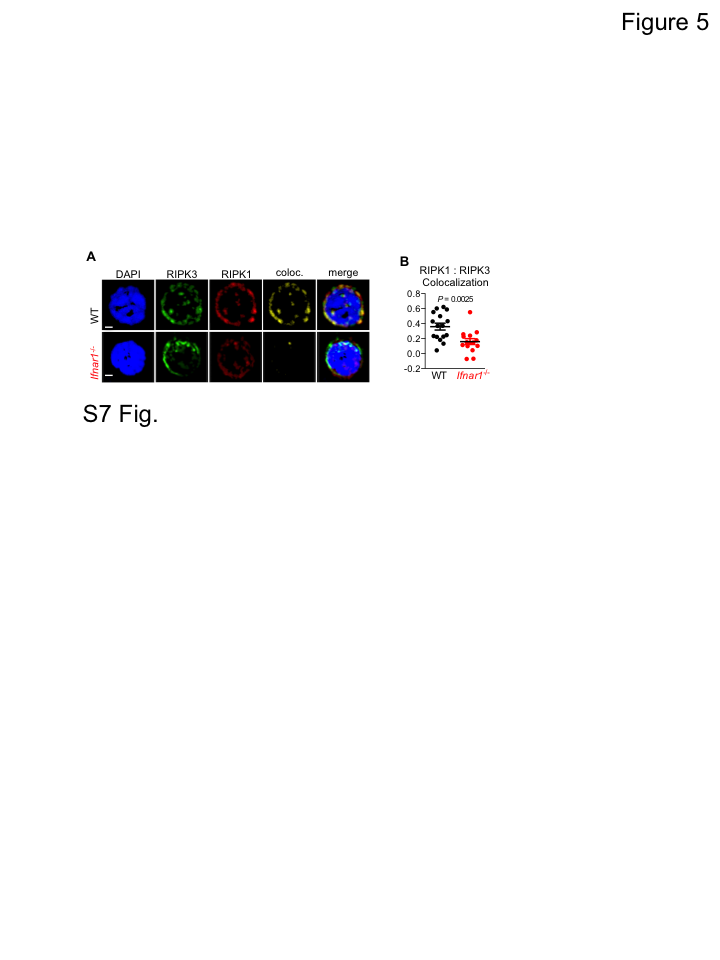

Supplement: S7 Fig — (A) Representative micrographs of RIPK3, RIPK1, and DAPI staining in HSPCs sorted from IOE-infected WT and Ifnar1-/- mice 7 d.p.i. Scale bar = 1μm. (B) Pearson’s coefficient of RIPK1 and RIPK3 colocalization. n = 15–16 cells analyzed/group. (TIFF) [file ppat.1007234.s007.tiff]

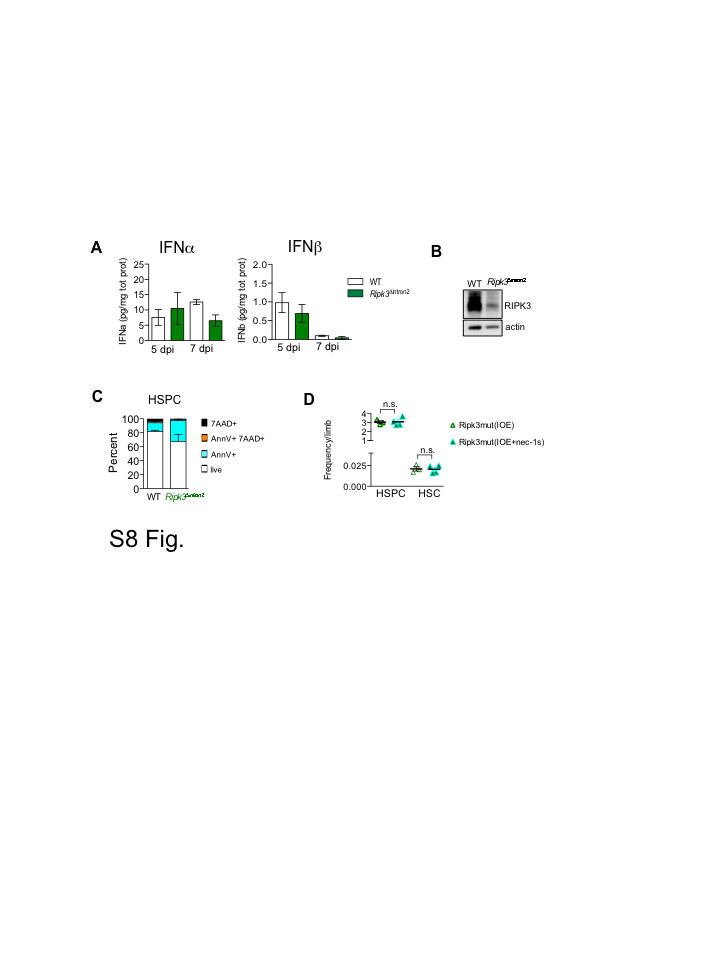

Supplement: S8 Fig — (A) IFNα and IFNb production in BM during IOE infection in WT and Ripk3Δintron2 mice at the indicated times post-infection. n = 3–4 mice/group. (B) RIPK3 detection in BM cell lysates from WT and Ripk3Δintron2 mice. (C) HSPC cell death (Annexin V and 7AAD staining) in WT and Ripk3Δintron2 mice on day 7 post-IOE infection. (D) Ripk3Δintron2 mice infected with IOE were treated with Nec-1s. Absolute frequencies of HSPCs and HSCs are shown in Veh-treated and Nec-1s treated mice. (TIFF) [file ppat.1007234.s008.tiff]
